# Evidence on treat to target strategies in polymyalgia rheumatica and giant cell arteritis: a systematic literature review

**DOI:** 10.1093/rheumatology/kead471

**Published:** 2023-09-06

**Authors:** Elvis Hysa, Milena Bond, Lisa Ehlers, Dario Camellino, Louise Falzon, Christian Dejaco, Frank Buttgereit, Daniel Aletaha, Andreas Kerschbaumer

**Affiliations:** Laboratory of Experimental Rheumatology and Academic Division of Clinical Rheumatology, Department of Internal Medicine, San Martino Polyclinic, University of Genoa, Genoa, Italy; Department of Rheumatology, Hospital of Bruneck (ASAA-SABES), Teaching Hospital of the Paracelsius Medical University, Bruneck, Italy; Department of Rheumatology and Clinical Immunology, Charité – Universitätsmedizin Berlin, Corporate Member of Freie Universität Berlin and Humboldt-Universität zu Berlin, Berlin, Germany; Division of Rheumatology, Local Health Trust 3, Genoa, Italy; Health Economics and Decision Science, School of Health and Related Research, University of Sheffield, Sheffield, England; Department of Rheumatology, Hospital of Bruneck (ASAA-SABES), Teaching Hospital of the Paracelsius Medical University, Bruneck, Italy; Department of Rheumatology, Medical University Graz, Graz, Austria; Department of Rheumatology and Clinical Immunology, Charité – Universitätsmedizin Berlin, Corporate Member of Freie Universität Berlin and Humboldt-Universität zu Berlin, Berlin, Germany; Division of Rheumatology, Department of Medicine III, Medical University of Vienna, Vienna, Austria; Division of Rheumatology, Department of Medicine III, Medical University of Vienna, Vienna, Austria

**Keywords:** GCA, PMR, treat to target, T2T

## Abstract

**Objectives:**

To inform an international task force about current evidence on Treat to Target (T2T) strategies in PMR and GCA.

**Methods:**

A systematic literature research (SLR) was conducted in Medline, EMBASE, Cochrane Library, clinicaltrials.gov from their inception date to May 2022, and in the EULAR/ACR abstract database (2019–2021). Randomised clinical trials (RCTs) and non-randomised interventional studies published in English and answering at least one of the eleven PICO questions on T2T strategies, treatment targets and outcomes, framed by the taskforce, were identified. Study selection process, data extraction and risk of bias assessment were conducted independently by two investigators.

**Results:**

Of 7809 screened abstracts, 397 were selected for detailed review and 76 manuscripts were finally included (31 RCTs, eight subgroup/exploratory analyses of RCTs and 37 non-randomised interventional studies). No study comparing a T2T strategy against standard of care was identified. In PMR RCTs, the most frequently applied outcomes concerned treatment (90.9% of RCTs), particularly the cumulative glucocorticoids (GC) dose and GC tapering, followed by clinical, laboratory and safety outcomes (63.3% each). Conversely, the most commonly reported outcomes in RCTs in GCA were prevention of relapses (72.2%), remission as well as treatment-related and safety outcomes (67.0% each).

**Conclusions:**

This SLR provides evidence and highlights the knowledge gaps on T2T strategies in PMR and GCA, informing the task force developing T2T recommendations for these diseases.

Rheumatology key messages

- In PMR studies, glucocorticoid tapering and discontinuation were the most commonly used outcomes.
- In GCA trials, disease activity parameters were prioritized as endpoints.
- No randomized controlled trial investigated a treat to target strategy in PMR and GCA yet.

## Introduction

PMR and GCA are overlapping inflammatory rheumatic disorders of the elderly [1–3]. Duration of glucocorticoid (GC) treatment and/or use of immunosuppressive drugs varies considerably among patients; however, many people with PMR or GCA are treated with GC for several years, particularly those with recurrent relapses [4]. Once remission has been achieved, an important goal is to minimize treatment toxicity and to balance dose reduction against the risk of relapse [5].

The treat to target (T2T) approach, implemented in several disciplines of medicine, has also been adopted in rheumatology. T2T recommendations are currently available for RA, PsA, axial spondylitis (axSpA) and SLE [6–8]. In RA, PsA and axSpA, regular monitoring with the aim to achieve a specific treatment target, and modification of treatment when the target has not been reached resulted in better clinical and structural outcomes than a conventional treatment strategy [9–13].

Although much progress has been made in the management of PMR and GCA, new unmet needs have emerged in terms of patients’ stratification, development of relevant treatment targets, and prevention of disease- and treatment-related complications. The objective of the present systematic literature review (SLR) was to inform an international task force developing new T2T recommendations in PMR and GCA about the evidence on treatment targets and outcomes in these conditions [14].

## Methods

This SLR was performed according to the Preferred Reporting Items for Systematic Reviews and Meta-Analyses (PRISMA) statement checklist [15]. At the first (virtual) meeting, the scientific committee. agreed on eleven key questions relevant to T2T in PMR and GCA (Table 1). The key clinical questions were eventually rephrased in the PICO format (Patients, Intervention, Comparator or Control, Outcome) which served as the basis for the SLR. A detailed description of the PICOs is depicted in Table 1.

**Table 1.** Clinical key questions and PICOs used for the systematic literature review

**Clinical key questions agreed upon by the scientific committee:** What are the treatment targets and outcomes in GCA/PMR, and how can they be measured (imaging, lab parameters, clinical, PRO)? Is coming off GC a treatment target in GCA/PMR, and how quickly should it be achieved? What should be the frequency of monitoring disease state/adapting therapy? How fast and to what extent should disease activity change before requiring treatment modification? How do comorbidities influence T2T outcomes in GCA/PMR? What are comorbidities related to uncontrolled disease activity? Do targets need to be adapted based on the presence of comorbidities? Is residual disease activity acceptable, and to what extent? How can reaching disease targets, reducing/preventing treatment side effects, and long-term consequences of disease be balanced in GCA/PMR? What is more important: control of disease activity or prevention of treatment-related adverse effects? Can treatment success be predicted? What are the predictors of successful treatment reduction (e.g. duration on target)? Do treatment targets differ over time (early vs. established disease)?
**PICO questions used for the systematic literature review:** In GCA (P1) or PMR (P2), what outcomes and treatment targets (O) have been used in clinical studies (I/C)? In GCA (P1) or PMR (P2), what is the outcome (O) applying specific treatment target(s) (I) as compared with standard of care/no target (C)? In GCA (P1) or PMR (P2), what is the outcome (O) of stopping GC (I) as compared with continuing GC (C) after achieving a target (e.g. remission)? In GCA (P1) or PMR (P2), what is the outcome (O) of a rapid tapering of GC (I) as compared with a slower tapering of GC (C) after achieving a target (e.g. remission)? In GCA (P1) or PMR (P2), what is the outcome (O) by monitoring disease state (I1) OR treatment AEs (I2) OR disease damage (I3) OR comorbidities (I4) with method/frequency A as compared with method/frequency B (C1) or no monitoring (C2)? In GCA (P1) or PMR (P2), what is the outcome/treatment target (O) in case of presence (I) *vs* absence of comorbidities (C)? In GCA (P1) or PMR (P2), what is the outcome (O) related to complete control of inflammation (I) compared with residual inflammation (C)? In GCA (P1) or PMR (P2), what is the outcome (O) using predictor A (I) *vs* predictor B (C1) or no predictor (C2)? In GCA (P1) or PMR (P2), what is the outcome (O) of treatment reduction (I) *vs* maintenance (C)? In GCA (P1) or PMR (P2), is achieving the target with the first treatment strategy (I) superior in achieving drug-free-remission or treatment-tapering (O) compared with multiple treatment switches (C)? In GCA (P1) or PMR (P2), do patients with early disease (I) differ from patients with established disease (C) in achieving treatment targets (O)?

AEs: adverse events; PICO: patients, intervention, comparator, outcome; T2T: treat-to-target.

An experienced librarian (L.F.) developed the search strategy: Ovid Medline, EMBASE, the Cochrane Library and Epistemonikos were searched from their inception date until 13 March 2021 (Supplementary Table S1, available at *Rheumatology* online). An update of the SLR was performed on 3 May 2022. A manual search of abstracts from ACR and EULAR meetings from 2019 to 2021 (grey literature) was conducted. The SLR incorporated studies published solely as conference abstracts, but in cases where the related abstract's full text was accessible, only the latter was considered. Additional articles were retrieved searching the reference list of original and review articles and by contacting experts in the field.

All identified citations were downloaded to the Covidence software (Veritas Health Innovation, Australia), and duplicates were removed. Four researchers (E.H., M.B., L.E. and D.C.) conducted the SLR under the supervision of the methodologists (A.K. and D.A.). D.C. and L.E. independently performed screening and selection of articles but, due to the COVID-19 pandemic, they were not able to continue with the project. Therefore, the subsequent phases of the process (i.e. data extraction, data synthesis and quality appraisal) were performed by E.H. and M.B. Discordances between reviewers were discussed until agreement was achieved. When consensus was not achieved, one of the methodologists was consulted for a final decision.

We included full articles or research letters of interventional studies [randomized clinical trials (RCTs) as well as non-randomised interventional studies including >20 PMR and/or GCA patients (all subtypes)], published in English, and with no age restriction. Studies further required to have a control group receiving either placebo or an active treatment. Study details and results were extracted independently by E.H. and M.B. using a standardised data extraction sheet. Items of interest were: (i) population type (PMR, GCA with cranial and or large vessel involvement, PMR/GCA overlap) and demographics; (ii) number of patients included and proportion of those randomized to/receiving treatment; (iii) intervention and control treatment; (iv) outcomes and treatment targets; (v) strategies to monitor disease activity, adverse events (AEs) and comorbidities; (vi) predictors of disease course; (vii) the effect of different treatment regimens and (viii) the prognostic role of early *vs* established disease on outcomes.

Risk of bias (RoB) was assessed at study level (eventually considering multiple publications from one study) using the Cochrane Collaborations Risk of Bias tool for RCTs and the ROBINS-I tool for non-randomised interventional studies [16–18]. Due to the heterogeneity of the available studies, no meta-analysis was performed, and results are reported separately for each study.

The contents of this SLR were presented during the face-to-face meeting of the scientific committee and the task force in June 2022 and provided the scientific basis for the T2T recommendations in PMR and GCA [14].

## Results

### Included studies

The search identified 7809 references. In total, 76 of them were finally included in our SLR: 31 RCTs, eight post-hoc analyses of RCTs and 37 non-randomised interventional studies (see PRISMA flowchart in Supplementary Fig. S1, available at *Rheumatology* online). Several articles contributed data to more than one PICO: 73 articles (96.0%; 31 RCTs, eight post-hoc analyses of RCTs and 34 non-randomised studies) were assigned to PICO 1, one non-randomized study (1.3%) to PICO 6, 15 (19.7%; 2 RCTs, two post-hoc analyses, 11 non-randomized interventional studies) to PICO 8 and three (3.9%; one RCT sub-analysis and two non-randomized studies) to PICO 11. For the remaining PICOs (PICOs 2–5, 7, 9 and 10) no evidence was found.

Full data on quality assessment for RCTs and non-randomized interventional studies are depicted in Supplementary Table S2 and S3 (available at *Rheumatology* online), respectively.

### Outcomes and treatment targets in polymyalgia rheumatica (PICO 1)

Twenty articles, 11 RCTs and nine non-randomized interventional studies were assigned to PICO 1 concerning outcomes and treatment targets (PICO 1).

#### Randomized controlled trials

**Figure 1.**
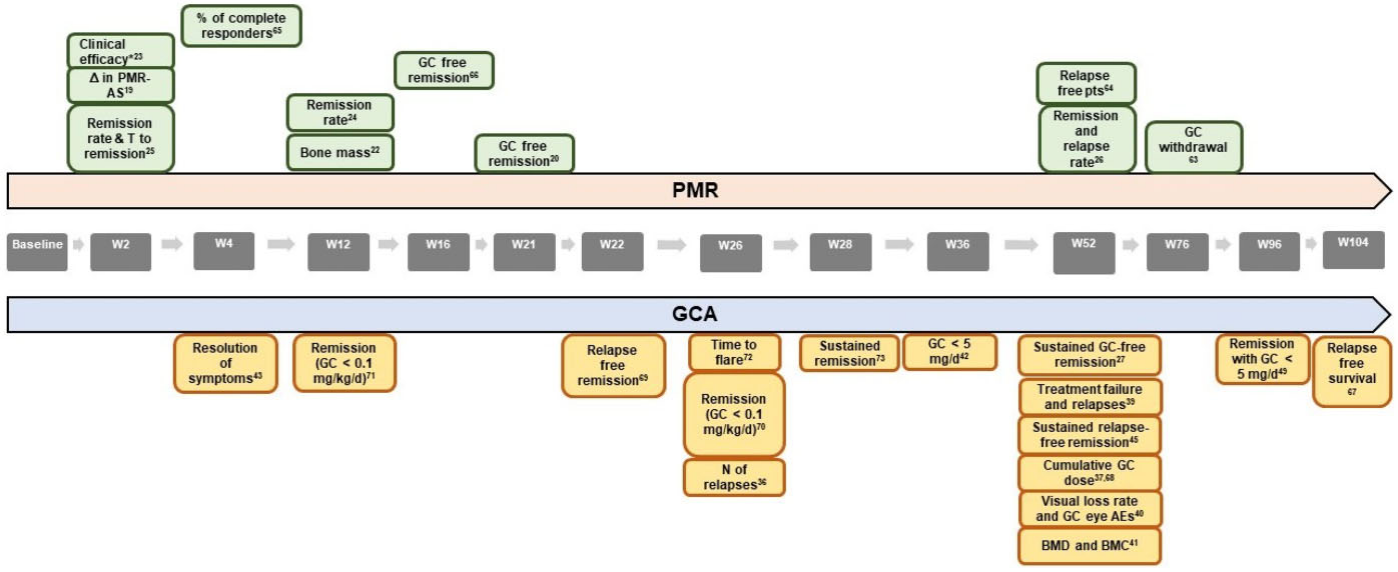
Primary outcomes used in PMR and GCA RCTs and time points of their assessment. AEs: adverse events; BMC: bone mineral content; BMD: bone mineral density; GC: glucocorticoids; N: number; PMR-AS: PMR activity score; pts: patients; T: time; W: week; Δ, change in. * Clinical efficacy was defined as (reduction of limb gridle pain, morning stiffness, ESR, CRP, fibrinogen, steroid dosage) at W2, 4, 6 and 12. Each reference is inserted as an exponential number

Six out of the eleven (54.5%) RCTs were considered to have low RoB. Unclear and high RoB were assigned to 5/11 (45.4%) and 1/11 (9.1%) studies, respectively as shown in Supplementary Table S2, available at *Rheumatology* online. In RCTs, clinical improvement was always part of the study outcomes (11/11, 100%), being included in either the definitions of remission (6/11, 54.5%) or relapse (5/11, 45.4%), or independently, in terms of resolution of PMR specific signs and symptoms (6/11, 54.5%). Table 2 and Fig. 1 depict a summary of the outcomes used in RCTs; full details are shown in Supplementary Table S4, available at *Rheumatology* online.

**Table 2.** Heat map of outcomes used in PMR RCTs

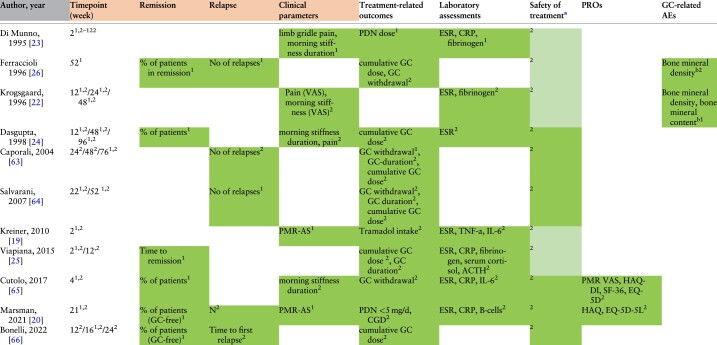

Overall, clinical and laboratory components were mainly used as outcomes and treatment targets. Assessment of clinical items was part of the study outcomes in all RCTs, in terms of the evaluation of either remission, relapse or resolution of specific signs and symptoms. Primary and secondary endpoints are expressed with numbers ‘1’ and ‘2’ inserted as exponential values in each outcome.

ACTH: adrenocorticotropic hormone; AE, adverse events; GC: glucocorticoids; IL-6: interleukin 6; Lab: laboratory component; MS: morning stiffness; N: number of relapses; PMR-AS: PMR activity score; PROs: patient reported outcomes; TNF-a: tumor necrosis factor alpha.

a Safety of treatment was summarized by extracting the data related to AEs ^b^assessed through DEXA.

Shading: AEs reported without being considered as a treatment target.

Outcomes related to PMR treatment (10 out of 11 studies, 90.9%) included the GC cumulative dose (7/10 studies, 70.0%), GC discontinuation (4/10, 40.0%), GC duration (3/10, 30.0%) or a specific GC target dose (2/10, 20.0%). A single study considered the cumulative intake of tramadol as a secondary outcome [19].

*Laboratory parameters (7 out of 11 studies, 63.3%).* Among laboratory parameters, the erythrocyte sedimentation rate (ESR, 7/7 studies, 100%), C-reactive protein (CRP, 3/7, 42.9%) and/or fibrinogen serum concentrations (3/7, 42.9%) were most frequently considered as outcomes. Interestingly, among RCTs assigned to PICO 1, CRP was always part of the laboratory assessments from 2010 onwards, while earlier studies primarily evaluated the ESR. Interleukin-6 (IL-6) was considered in 2/7 (28.6%) studies, serum tumour necrosis factor alpha (TNFα), cortisol and adrenocorticotropic hormone (ACTH) levels in one (14.3%) RCT each. One recent study, assessing the efficacy of rituximab in PMR patients, also investigated B-cell depletion as an outcome [20].

*Individual clinical parameters (6 out of 11 studies, 54.5%).* Morning stiffness was the most frequent clinical component (6/6, 100%), being evaluated either separately (4/6, 66.7%), or as part of a composite score (i.e. the PMR activity score [21] in 2/6 studies, 33.3%). With the exception of one study who measured the intensity of morning stiffness (through a scale from 0 to 3) [22], it was mainly the duration of stiffness that was taken into account. Pain was assessed in 3/6 (50.0%) studies through either the pain VAS scale [23, 24] (100 mm; 0 = best, 100 = worst) or a 0–3 Likert scale [22] (0 = best, 3 = worst).

*Safety of treatment (6 out of 11 studies, 54.5%).* All RCTs reported AEs, none of them considered them as a primary end point, but 6/11 (54.5%) included AEs as a secondary outcome. No study addressed the reduction of GC-related AEs as a specific outcome.

**Figure 2.**
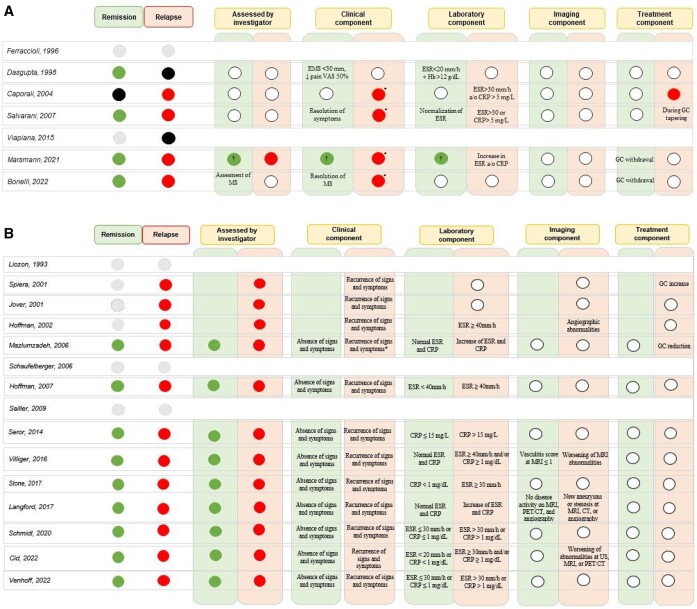
Components used in defining remission and relapse in (**A**) PMR RCTs# and (**B**) GCA RCTs#. Grey circle: Remission and/or relapse used as outcomes but not defined in the study methods; Green circle: remission (or specific components) defined in the study methods; Red circle: relapse (or specific components) defined in the study methods; White circle: component not part of the definition of remission/relapse. Black circle: remission/relapse not used as an outcome. CRP, C-reactive protein; CT, computerized tomography; EMS, early morning stiffness; ESR, erythrocyte sedimentation rate; GC, glucocorticoids; Hb, haemoglobin; MS, morning stiffness; MRI, magnetic resonance imaging; PET, positron emission tomography; US, ultrasound; VAS, visual analogue scale. *, signs and symptoms of active polymyalgia rheumatica; †, remission defined by PMR-AS (PMR Activity Score) <10; a/o, and/or. #Only RCTs considering remission and/or relapse as an outcome are listed. Overall, in (**A**) both remission and relapse were mainly defined as a combination of clinical and laboratory parameters. None of the studies defined sustained remission. In (**B**) relapse was defined as the return of signs and symptoms and/or an increase of ESR/CRP after reduction of prednisone dosage followed by an improvement of signs and symptoms when GC dosage was increased. Recurrence was defined as the reappearance of GCA signs and symptoms and/or increase of the inflammatory markers in a GCA patients not receiving GC therapy for at least 1 month. Overall, in GCA RCTs, both remission and relapse were mainly defined as a combination of clinical and laboratory parameters

*Remission (6 out of 11 studies, 54.5%).* Remission was evaluated as an outcome in 6/11 studies. Two of them referred to GC-free remission and one to the time to achieve remission. Remission was mostly defined by a combination of clinical and laboratory parameters, as shown in Fig. 2a. There was large heterogeneity between studies on when to measure remission, with a time span ranging from 2 weeks to 1 year after start of treatment [25, 26].

*Relapse (5 out of 11 studies, 45.4%).* Relapse was an outcome in 5/11 RCTs; one of these evaluated the time to first relapse. A relapse was always defined by a combination of clinical and laboratory parameters as shown in Fig. 2a.

*PROs (27.3%).* PROs were part of the outcome measures in only 3/11 studies. The health assessment questionnaire disability index (HAQ-DI) (2/3, 66.7%), the EuroQol-5 dimension (EQ-5D) (2/3, 66.7%), the 36-Item Short-Form Health Survey (SF-36) (1/3, 33.3%), and pain visual analogue scale (VAS; 100 mm; 0 = best, 100 = worst) (1/3, 33.3% each) were used.

*Other parameters.* Two studies considered bone mineral content (BMC) or bone mineral density (BMD) either as a primary [22] or secondary outcome [26].

### Outcomes and treatment targets in giant cell arteritis (PICO 1)

Forty-six articles were assigned to PICO 1: 18 RCTs, 20 non-randomised interventional studies and eight sub-analyses of RCTs. Among the latter, seven manuscripts were related to the GiACTA trial, a placebo-controlled phase III RCT to study the efficacy of tocilizumab in GCA [27] and one article was a post-hoc analysis of the phase II study on tocilizumab [28]. Among the eight papers related to GIACTA, the main publication analysed a number of pre-specified endpoints of the first double-blind phase of 52 weeks [27] whereas another reported the data from the second, open-label phase between weeks 52 and 104 [29]. The other six were subgroup analyses of the double-blind phase of GiACTA [30–35].

#### Randomized controlled trials

*Relapses (13/18, 72.2%):* In 3/13 RCTs (23%) and in 10/13 RCTs (77%), relapses were included as a primary or secondary end point, respectively. In 8/13 (61.5%) RCTs, the number of relapses was assessed whereas in 5/13 (38.5%) the time to first relapse was considered. All definitions of relapse used in these trials are summarized in Fig. 2b.

*Remission (12/18, 66.7%):* In 8 out of 12 RCTs (67.0%), remission was the primary end point. In seven (58.3%) and five RCTs (41.7%), remission combined with the achievement of a target GC dose ≤5 mg prednisone (PDN) equivalent per day or GC-free remission were used as outcomes, respectively. The time point when the achievement of remission was investigated was variable ranging from 3 to 24 months (See Table 3 for the time points of remission assessment, Fig. 2b for the definition of remission and Fig. 1 for the time points of evaluation of the primary endpoints in GCA).

**Table 3.** Heat map of outcomes used in GCA RCTs

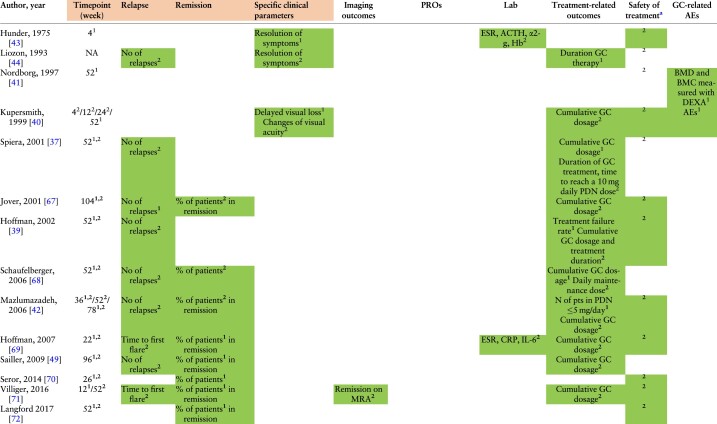

(continued)

**Table 3.** (continued)

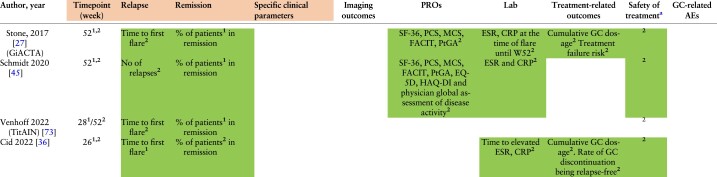

Overall, clinical and laboratory components were mainly used as outcomes and treatment targets. Assessment of clinical items was part of the study outcomes in 94.4% of the included RCTs, in terms of the evaluation of either remission, relapse or resolution of specific signs and symptoms.

Primary and secondary endpoints are expressed with numbers ‘1’ and ‘2’ inserted as exponential values in each outcome.

α2-g, alfa-2 globulins; ACTH, adrenocorticotropic hormone; AEs, adverse events; BMC, bone mineral content; BMD, bone mineral density; CRP, C-reactive protein; DEXA, dual-energy X-ray absorption; EQ-5D, EuroQol-5D; ESR, erythrocyte sedimentation rate; FACIT, Functional Assessment of Chronic Illness Therapy-Fatigue; GC, glucocorticoid; HAQ-DI, health assessment questionnaire disability index; IL-6, interleukin-6; MCS, Mental Component Summary scores and domains; MRA, magnetic resonance angiography; NA, not assessed; No, number; PCS, Physical Component Summary; PDN, prednisone; pts, patients; PtGA, Patient Global Assessment of Disease Activity; SF-36, 36-Item Short-Form Health Survey.

Shading: AEs reported without being considered part of the outcomes.

a safety of treatment was summarized by extracting the data related to AEs.

*Treatment-related outcomes (12/18, 66.7%):* In 3/12 RCTs, treatment related outcomes were the primary end point. The most frequently reported outcome was the cumulative GC dose (11/18, 61.1%), followed by the risk of treatment failure (2/18, 11.1%), GC discontinuation (1/18, 5.5%) [36] and the time required to reach a target prednisone dose of 10 mg/day (1/18, 5.5%) [37]. In two trials, treatment failure was defined as the inability to achieve remission by week 12 or the occurrence of a relapse between weeks 12 and 52 [38] or as two distinct relapses or persistence of disease activity after the first relapse, in spite of increment of the PDN dose by ≥10 mg [39].

*Safety of treatment (12/18, 66.7%):* All RCTs reported AEs, safety of treatment was specified as the primary end point in two trials [40, 41] and as a secondary end point in 10/18 (55%) RCTs. Among the RCTs considering AEs as secondary endpoints, two of them focused on GC-related Aes [42, 43]. The RCTs including safety as primary endpoints evaluated ocular complications [40], or the changes of bone mineral density (BMD), measured with dual-energy X-ray absorptiometry (DEXA) [41] after one year of GC treatment.

*Laboratory outcomes (27.8%):* Among laboratory outcomes, the most frequently reported tests were ESR (5/5, 100%) and CRP (4/5, 80.0%), followed by serum concentrations of IL-6 (1/5, 20.0%), alpha2-globulins (1/5, 20.0%) and fibrinogen (1/5, 20.0%).

*Specific clinical parameters, physician and patient-reported outcomes*: Among the three trials reporting clinical parameters separately from remission/relapses as endpoints, two of them (75.0%) focused on the resolutions of symptoms as a primary [43] or secondary outcome [44]. Another RCT (33.3%) considered the rate of delayed visual loss in the first year as a primary end point [40]. Additionally, two trials assessed PROs as secondary outcomes [31, 45]; one of them also reporting the physician global assessment [45].

PROs included in both studies were the following: the SF-36, the Physical Component Summary (PCS), the Mental Component Summary scores (MCS), the Functional Assessment of Chronic Illness Therapy (FACIT)-Fatigue and the Patient Global Assessment of Disease Activity (PtGA). Additional scales, included in the research work of Schmidt *et al.* [45], were the EuroQoL-5D, the EuroQoL-5D visual analogue score, the HAQ-DI, the Physician’s Global Assessment of Disease Activity and a numeric pain rating scale.

*Imaging outcomes (1/18, 5.5%):* One RCT included imaging as a secondary outcome: more specifically, remission was defined according to magnetic resonance angiography (MRA) score ≤1 (range of the score from 0 to 3 with 0 indicating no mural thickening/enhancement and 3 suggesting strong mural thickening and perivascular enhancement) [28].

Detailed information about the outcomes and the main findings of the included trials are detailed in Supplementary Table S5, available at *Rheumatology* online.

*PMR and GCA (mixed population).* Seven studies assessing PMR and GCA as a single group reported data for PICO 1: two RCTs and five non-randomized studies. Both RCTs (both with either unclear or high RoB) evaluated treatment-related outcomes in terms of GC cumulative dose, change in GC dose and GC duration. Safety was reported as a secondary outcome in both studies (100%), referring either to MTX [46] or azathioprine [47]. None of the RCTs assessed the reduction of GC-related side effects (see Supplementary Table S6, available at *Rheumatology* online, for details). Remission (defined as treatment discontinuation) and relapse (defined as recurrence of original symptoms and increase of ESR or CRP in patients still receiving GC) were investigated in only one study (50%) [46].

The summarized data on outcomes and treatment targets for non-randomized studies in PMR and GCA are reported in the Supplementary Boxes S1–S3, available at *Rheumatology* online.

### Impact of comorbidities on outcomes and treatment targets (PICO 6)

For GCA, only a single observational study provided evidence for PICO 6. In that study, the relapse rate of patients with biopsy-proven GCA was evaluated at one, two and five years. Hypertension (*P* = 0.007) and type 2 diabetes mellitus (*P* = 0.039) at the time of GCA diagnosis were associated with higher relapse rates, compared with those without these comorbidities. A higher proportion of patients with these comorbidities were present in the high relapse rate group (>0.5 relapses/year) compared with the group of no relapses or <0.5 relapses/year groups [48]. No data were available for patients with PMR.

### Predictors of outcomes (PICO 8)

*GCA.* Eleven studies on GCA, including two RCTs with two subgroup analyses of the GIACTA trial and seven non-randomised interventional studies, were assigned to PICO 8. Among data resulting from RCTs, female sex, an initial PDN-dosage equivalent ≤30 mg daily, worse pre-treatment PROs inherent to the perception of disease activity (measured with the PtGA), fatigue (FACIT-Fatigue) and general health status (SF-36 or EQ-5D scores), and increased ESR levels after achieving remission were associated with a higher risk of relapses (Table 4) [32, 33, 39, 49]. Another RCT on GCA, published only as a conference abstract, identified male gender and PMR symptoms at baseline as protective factors against a relapse [49].

**Table 4.** Predictors used in RCTs on GCA

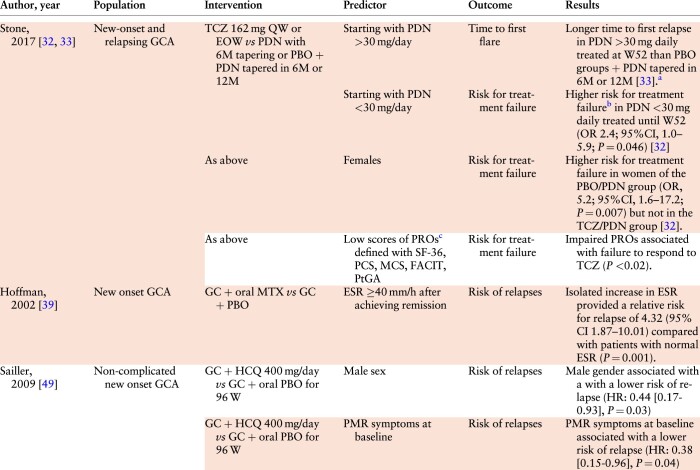

a These results are reported in the article as a Kaplan–Meier plot.

b Treatment failure defined as the inability to achieve remission by week 12 or the occurrence of a relapse between weeks 12 and 52.

c PROs were defined by standardised questionnaires self-reported by the patients.

EOW: every other week; FACIT: Functional Assessment of Chronic Illness Therapy-Fatigue; GC: glucocorticoid; HR: hazard ratio; M: month; MCS: Mental Component Summary scores and domains; OR: odds ratio; PBO: placebo; PCS: Physical Component Summary; PDN: prednisone; PtGA: Patient Global Assessment of Disease Activity; QW: every week; SF-36: 36-Item Short-Form Health Survey; TCZ: tocilizumab.

The systematic search for RCTs investigating prognostic factors for PMR did not yield any data.

Predictors of outcomes in non-randomized studies on PMR and GCA are reported, in summary, in Supplementary Boxes S4 and S5, available at *Rheumatology* online.

### Outcomes in early *vs* established disease (PICO 11)

Three studies on GCA reported data for PICO 11, a post-hoc analysis of GiACTA [30] and two other non-randomized interventional studies [50, 51]. In the open-label extension of GiACTA, the number of flares at 3 years was compared between new-onset *vs* relapsing GCA. The authors reported that the relapse rate did not differ between these groups when the same treatment arm was analysed (TCZ every week, TCZ every other week or PBO). The disappearance of GCA signs and symptoms as well as changes in acute phase reactants after treatment with TCZ + GC or GC monotherapy were assessed in another observational study [50]. Among those who received TCZ, no difference was observed between patients with new-onset and established disease (defined as disease duration >6 months) concerning these outcomes. In another study of patients treated with leflunomide or MTX upon the occurrence of a (first) relapse, the rate of subsequent relapses was similar between groups with early and late GCA defined as disease duration less or more than one month, respectively [51].

No data were available from PMR studies on this PICO.

## Discussion

In studies on PMR, GC-related outcomes (i.e. cumulative GC dose, GC discontinuation, GC duration, or a specific GC target dose) were the most common treatment targets (90.9%); remission and relapse were applied in only half of studies. In contrast, prevention of relapses, achievement of remission, and cumulative GC dosage were the most frequent targets in trials of GCA.

The preference of GC-related outcomes in PMR and outcomes related to disease activity in GCA remains subject to speculation. An explanation could be the fact that disease activity in PMR is difficult to assess because of the presence of comorbidities affecting or mimicking PMR symptoms [52, 53]. Another reason could be the observation that PMR does not cause long-term organ damage by itself (while certainly impacting quality of life when active) whereas the majority of patients experience AEs related to long-term GC therapy [54]. The reduction of GCs is therefore an important therapeutic goal in PMR. In GCA, disease activity potentially leads to vascular and organ damage, when not adequately treated [55].

In order to personalize treatment, it would be desirable to stratify patients according to disease severity, comorbidities and risk of developing GC-related toxicities. Unfortunately, data on this aspect are scarce, especially for PMR, whose predictors of clinical response (lower weight, age >60 years at diagnosis, extracapsular inflammatory pattern in MRI and increased musculoskeletal uptake in PET/CT) derive only from non-randomized studies of unclear or high risk of bias [56–59]. In GCA, female sex, a PDN-equivalent starting dosage <30 mg/day, absence of PMR symptoms, impaired pre-treatment PROs, and persistently raised acute phase reactants after achieving clinical remission predicted a higher relapse rate [32, 33, 39, 49].

We acknowledge that our SLR was limited to RCTs and non-randomised interventional studies, while studies without a control group were excluded. Therefore, some possible predictors might have been missed. Data from observational studies (not meeting our inclusion criteria) for example identified female sex, high acute-phase reactants levels, peripheral arthritis, higher starting GC dosage and fast tapering as possible predictors of PMR relapse and need for prolonged GC treatment [4]. In fact, despite mentioning that the quality of the evidence of the studies was low to moderate, female sex, high acute-phase reactants levels and peripheral arthritis are also reported as prognostic factors in the 2015 EULAR/ACR recommendations for the management of PMR [60]. In addition, a recent meta-analysis showed that female sex and large-vessel involvement are predictors of relapse in GCA [61]. Moreover, due to the inclusion criteria used in the present SLR, specific risk factors for ischaemic neuro-ophthalmic complications were not captured (i.e. jaw claudication, diplopia and temporal artery abnormalities as reported in the British Society of Rheumatology guidelines of GCA management [62], although originating mostly from observational studies).

We did not identify a study testing a T2T strategy in PMR and/or GCA. An obstacle to conduct such a trial might be the absence of internationally recognised remission criteria, which have been defined as the most relevant target for these diseases. Optimally, PMR and GCA trials adopting a T2T approach may be important for individualizing treatment, reducing side effects, and enhancing quality of life. Frequent monitoring aids in early detection of flares, enabling timely intervention to prevent severe exacerbations [10, 11, 13]. Moreover, this approach would foster standardized care, gather valuable research data, and support evidence-based decision-making, ultimately leading to improved long-term outcomes. Furthermore, the utilization of a T2T strategy calls for the introduction of new therapeutic interventions targeting individuals who exhibit limited responsiveness to the initial treatment regimen. We anticipate that this SLR and the T2T recommendations will stimulate further research in this regard [14].

We did not include studies without a control group in our SLR, which might be seen as a limitation; however, our objective was to identify targets and outcomes that might be valuable for a T2T strategy. Studies without an intervention were therefore considered less relevant for our purpose. Titles and abstract screening were performed by different fellows than data extraction and quality assessment; this is certainly not the standard approach and was a consequence of changed duties and personal developments during the COVID-19 crisis. However, every step was conducted under the supervision of the methodologists who guarantee the homogeneity of the different steps of this SLR.

In summary, our SLR synthetized the outcomes and treatment targets used in PMR and GCA RCTs and non-randomised interventional studies. GC cumulative dose and tapering were mostly considered as a target in PMR, while prevention of relapses and achievement of remission were mainly applied in GCA. This SLR informed the international task force developing the T2T recommendations for PMR and GCA.

## Supplementary Material

kead471_Supplementary_Data

## Data Availability

Data used in the preparation of this review are available on request from the corresponding author. E.H., M.B., L.E. and D.C. have full access to all the data in the study and take responsibility for the integrity of the data and the accuracy of the data analysis.
