# Supplementary material for: Evidence on treat to target strategies in polymyalgia rheumatica and giant cell arteritis: a systematic literature review
Source: Rheumatology (Oxford). 2023 Sep 6;63(2):285–97. doi: 10.1093/rheumatology/kead471 (PMC10836985; doi:10.1093/rheumatology/kead471)
Supplement: kead471_Supplementary_Data [file kead471_supplementary_data.docx]

**Supplementary Table S1.** Key words used for the systematic literature search.

| **MEDLINE**  1 Polymyalgia Rheumatica/  2 polymyal$.tw.  3 Giant Cell Arteritis/  4 Aortitis/ (1643)  5 ((giant cell or temporal or isolated or cranial or large vessel) adj2 (arterit$ or aortit$ or vasculit$)).tw.  6 horton$ disease.tw.  7 (lvv or gca).tw.  8 or/1-7  9 randomized controlled trial.pt.  10 controlled clinical trial.pt.  11 randomized.ab.  12 placebo.ab.  13 drug therapy.fs.  14 randomly.ab.  15 trial.ab.  16 groups.ab.  17 exp cohort studies/  18 observational study.pt.  19 (cohort$ or prospective$ or observational or longitudinal or follow-up).tw.  20 or/9-19  21 8 and 20  22 exp animals/ not humans.sh.  23 21 not 22  **The Cochrane Library**  #1 MeSH descriptor: [Polymyalgia Rheumatica] this term only  #2 polymyal*:ti,ab  #3 MeSH descriptor: [Giant Cell Arteritis] this term only  #4 MeSH descriptor: [Aortitis] this term only  #5 (("giant cell" OR temporal OR isolated OR cranial OR "large vessel") NEAR/2 (arteri* OR aortit* OR vasculit*)):ti,ab  #6 "horton disease":ti,ab OR "hortons disease":ti,ab  #7 (lvv OR GCG):ti,ab  #8 #1 OR #2 OR #3 OR #4 OR #5 OR #6 OR #7  **EMBASE**  22. #8 AND #20 AND ([article]/lim OR [article in press]/lim OR [review]/lim) AND [humans]/lim  #21. #8 AND #20  #20. #9 OR #10 OR #11 OR #12 OR #13 OR #14 OR #15 OR #16 OR #17 OR #18 OR #19  #19. cohort*:ab,ti OR prospective*:ab,ti OR observational:ab,ti OR longitudinal:ab,ti OR 'follow up':ab,ti  #18. 'longitudinal study'/exp OR 'prospective study'/de  #17. 'crossover procedure'/de  #16. 'single‐blind procedure'  #15. crossover*:ab,ti OR 'cross over*':ab,ti  #14. placebo*:ab,ti  #13. (doubl* NEAR/2 blind*):ab,ti  #12. allocat*:ab,ti  #11. trial:ti  #10. 'randomized controlled trial'/exp  #9. random*:ab,ti  #8. #1 OR #2 OR #3 OR #4 OR #5 OR #6 OR #7  #7. lvv:ab,ti OR gca:ab,ti  #6. 'horton disease':ab,ti OR 'hortons disease':ab,ti  #5. (('giant cell' OR temporal OR isolated OR cranial OR 'large vessel') NEAR/2 (arteri* OR aortit* OR vasculit*)):ab,ti  #4. 'aortitis'/de  #3. 'giant cell arteritis'/de  #2. polymyal*:ab,ti  #1. 'rheumatic polymyalgia'/de    **EPISTEMONIKOS**  #1 (title:(Polymyalg*) OR abstract:(Polymyalg*))  #2 title:((("giant cell" OR temporal OR isolated OR cranial OR "large vessel") AND (arteri* OR aortit* OR vasculit*))) OR abstract:((("giant cell" OR temporal OR isolated OR cranial OR "large vessel") AND (arteri* OR aortit* OR vasculit*))))  #3 (title:("horton disease" OR "hortons disease") OR abstract:("horton disease" OR "hortons disease"))  #4 #1 OR #2 OR #3 |
| --- |

**Supplementary Table S2.** Results of quality assessment of RCTs on PMR and/or GCA.

**Legend**. PMR, polymyalgia rheumatica; GCA, giant cell arteritis; RoB, Risk of Bias; MTX, methotrexate; PDN, prednisone, GC, glucocorticoid; AEs, adverse events.

| **Author, year** | **Domain 1: Randomization process** | **Domain 2:**  **Effect of assignment to intervention** | **Domain 3:**  **Effect of adhering to intervention** | **Domain 4: Missing outcome data** | **Domain 5: Measurement of the outcomes** | **Domain 6: Selection of the reported result** | **Overall RoB** |
| --- | --- | --- | --- | --- | --- | --- | --- |
| **PMR** | | | | | | | |
| **Bonelli, 2022^1^** | Low RoB | Low RoB | Low RoB | Low RoB | Low RoB | Low RoB | Low RoB |
| **Caporali, 2004^2^** | Low RoB | Low RoB | Low RoB | Low RoB | Low RoB | Low RoB | Low RoB |
| **Cutolo, 2017^3^** | Low RoB | Low RoB | Low RoB | Low RoB | Low RoB | Low RoB | Low RoB |
| **Dasgupta, 1998^4^** | Low RoB | Low RoB | Low RoB | Low RoB | Low RoB | Low RoB | Low RoB |
| **Marsman, 2021^5^** | Low RoB | Low RoB | Low RoB | Low RoB | Low RoB | Low RoB | Low RoB |
| **Salvarani, 2007^6^** | Low RoB | Low RoB | Low RoB | Low RoB | Low RoB | Low RoB | Low RoB |
| **Di Munno, 1995^7^** | Unclear RoB:  Insufficient explanations about allocation concealment | Low RoB | Low RoB | Low RoB | Low RoB | Low RoB | Unclear RoB |
| **Kreiner, 2010^8^** | Low RoB | Low RoB | Low RoB | Low RoB | Low RoB | Low RoB | Low RoB |
| **Krogsgaard, 1996^9^** | Unclear RoB:  Insufficient explanations about allocation concealment | Low RoB | Low RoB | Low RoB | Low RoB | Low RoB | Unclear RoB |
| **Viapiana, 2015^10^** | Unclear RoB:  Insufficient explanations about allocation concealment | Low RoB | Low RoB | Low RoB | Low RoB | Low RoB | Unclear RoB |
| **Ferraccioli, 1996^11^** | High RoB:  Randomization process not defined | High RoB:  Heterogeneity in treatment (different GC dosages: MTX + PDN 25 vs PDN 15 mg) | Low RoB | Low RoB | High RoB:  Absence of pre-specified outcomes | Low RoB | High RoB |
| **GCA** | | | | | | | |
| **Stone 2017**  **(GiACTA)^12^** | Low RoB | Low RoB | Low RoB | Low RoB | Low RoB | Low RoB | Low RoB |
| **Stone 2021^13^** | Low RoB | Low RoB | Low RoB | Low RoB | Low RoB | Low RoB | Low RoB |
| **Stone 2021^14^** | Low RoB | Low RoB | Low RoB | Low RoB | Low RoB | Low RoB | Low RoB |
| **Strand 2019^15^** | Low RoB | Low RoB | Low RoB | Low RoB | Low RoB | Low RoB | Low RoB |
| **Unizony 2021^16^** | Low RoB | Low RoB | Low RoB | Low RoB | Low RoB | Low RoB | Low RoB |
| **Stone 2019^17^** | Low RoB | Low RoB | Low RoB | Low RoB | Low RoB | Low RoB | Low RoB |
| **Spiera 2021^18^** | Low RoB | Low RoB | Low RoB | Low RoB | Low RoB | Low RoB | Low RoB |
| **Mohan 2020^19^** | - | - | - | - | - | - | Quality assessment not performed: paper published as a conference abstract |
| **Cid 2022^20^** | Low RoB | Low RoB | Low RoB | Low RoB | Low RoB | Low RoB | Low RoB |
| **Mazlumazadeh 2006^21^** | Low RoB | Low RoB | Low RoB | Low RoB | Low RoB | Low RoB | Low RoB |
| **Spiera 2001^22^** | Low RoB | Low RoB | Low RoB | Low RoB | Low RoB | Low RoB | Low RoB |
| **Villiger 2016^23^** | Low RoB | Low RoB | Low RoB | Low RoB | Low RoB | Low RoB | Low RoB |
| **Reichenbach 2018^24^** | Low RoB | Low RoB | Low RoB | Low RoB | Low RoB | Low RoB | Low RoB |
| **Langford 2017^25^** | Low RoB | Low RoB | Low RoB | Low RoB | Low RoB | Low RoB | Low RoB |
| **Seror 2014^26^** | Low RoB | Low RoB | Low RoB | Low RoB | Low RoB | Low RoB | Low RoB |
| **Hoffman 2002^27^** | Low RoB | Low RoB | Low RoB | Low RoB | Low RoB | Low RoB | Low RoB |
| **Jover 2001^28^** | Low RoB | Low RoB | Low RoB | Low RoB | Low RoB | Low RoB | Low RoB |
| **Hoffman 2007^29^** | Low RoB | Low RoB | Low RoB | Low RoB | Low RoB | Low RoB | Low RoB |
| **Sailler 2009^30^** | - | - | - | - | - | - | Quality assessment not performed: paper published as a conference abstract |
| **Venhoff 2022 (TitAIN)^31^** | - | - | - | - | - | - | Quality assessment not performed: paper published as a conference abstract |
| **Schmidt 2020^32^** | Low RoB | Low RoB | Low RoB | Unclear RoB:  Small percentage of patients (17%) completing the study | Low RoB | Low RoB | Unclear RoB |
| **Nordborg 1997^33^** | Unclear RoB:  Insufficient explanations about allocation concealment | Low RoB | Low RoB | Low RoB | Low RoB | Low RoB | Unclear RoB |
| **Liozon 1993^34^** | High RoB:  Randomization process not defined, allocation concealment and blinding not specified | Low RoB | Low RoB | High RoB:  Premature termination of the study due to AEs | Low RoB | High RoB:  Selective reporting outcomes | High RoB |
| **Schaufelberger 2006^35^** | High RoB:  Randomization process not defined | Low RoB | Low RoB | High RoB:  Premature termination of the study due to AEs | Low RoB | High RoB:  Selective reporting outcomes | High RoB |
| **Kupersmith 1999^36^** | High RoB:  Randomization process not defined | High RoB:  Heterogeneous treatment  (Elevated variability in the initial treatment with GC from 40 to 1000 mg) | Low RoB | Low RoB | Low RoB | Low RoB | High RoB |
| **Hunder 1975^37^** | High RoB:  Randomization process not defined | Unclear RoB:  Not clear the allocation concealment, sequence generation nor blinding | Low RoB | Low RoB | High RoB:  Absence of pre-specified outcomes | High RoB:  Selective reporting outcomes | High RoB |
| PMR and GCA | | | | | | | |
| **Van der Veen 1996^38^** | Unclear RoB:  Non-homogeneus population.  Randomization not specified | Low RoB | Low RoB | Low RoB | Unclear RoB:  Outcomes not well pre-specified | Low RoB | Unclear RoB |
| **De Silva 1986^39^** | High RoB:  Randomization process not defined | High RoB:  Not clear the allocation concealment, sequence generation nor blinding | Low RoB | Low RoB | Low RoB | Low RoB | High RoB |

**Supplementary Table S3.** Quality assessment of non-randomised studies of intervention performed with ROBINS-I.

**Legend.** Domains: D1: Bias due to confounding; D2: Bias due to selection of participants; D3: Bias in classification of interventions; D4: Bias due to deviations from intended interventions; D5: Bias due to missing data; D6: Bias in the measurement of outcomes; D7: Bias in the selection of the reported results. Abbreviations: LV, large vessel; GC, glucocorticoids; CV, cardiovascular; BMI, body mass index; FR, rheumatoid factor; ACPA, anti-citrullinated peptide antibodies; [MTX,](https://www.ncbi.nlm.nih.gov/pmc/articles/PMC2859449/) methotrexate; HD, high dosage; cs/bDMARDs, conventional synthetic/biological disease modifying anti-rheumatic drugs; TCZ, tocilizumab; ESR, erythrocyte sedimentation rate; CRP, C-reactive protein; LEF, leflunomide; PET, positron emission tomography; ABA, abatacept.

| **Author, year** | **D1** | **D2** | **D3** | **D4** | **D5** | **D6** | **D7** | **Overall** |
| --- | --- | --- | --- | --- | --- | --- | --- | --- |
| **PMR** | | | | | | | | |
| Gabriel, 1997^40^ | High RoB:  Baseline population characteristic not well described | Low RoB | Low RoB | Low RoB | Low RoB | Low RoB | Low RoB | High RoB |
| Myklebust, 2001^41^ | High RoB:  LV involvement not considered; GC dose highly heterogeneous | Low RoB | Low RoB | Low RoB | Low RoB | Low RoB | Low RoB | High RoB |
| Maradit Kremers, 2007^42^ | Unclear RoB:  Concomitant CV risk factors and concomitant medications not considered | Low RoB | Low RoB | Low RoB | Low RoB | Low RoB | Low RoB | Unclear RoB |
| Cimmino, 2008^43^ | Unclear RoB:  Population baseline characteristics not well described | Low RoB | Low RoB | Low RoB | Unclear RoB: Missing data | Low RoB | Low RoB | Unclear RoB |
| Cimmino, 2011^44^ | Low RoB | Low RoB | Low RoB | Low RoB | Low RoB | High RoB:  “low weight” not defined in methods; BMI not assessed | Low RoB | High RoB |
| Benucci, 2015^45^ | Low RoB | Low RoB | Low RoB | Low RoB | Low RoB | Time points of predefined outcomes not well defined | Selective reporting outcomes | Unclear RoB |
| Mackie, 2015^46^ | Low RoB | Low RoB | Low RoB | Low RoB | Low RoB | Low RoB | Low RoB | Low RoB |
| Charpentier, 2018^47^ | Unclear RoB:  short follow-up, patients with presence of FR and ACPA not excluded | Low RoB | Low RoB | Low RoB | Low RoB | Low RoB | Low RoB | Unclear RoB |
| Quartuccio, 2018^48^ | Unclear RoB:  Compared populations not homogeneous | Low RoB | Low RoB | Low RoB | Low RoB | Low RoB | Low RoB | Unclear RoB |
| Giollo, 2019^49^ | Unclear RoB:  Method used to define population not validated | Low RoB | Low RoB | Low RoB | Low RoB | Low RoB | Low RoB | Unclear RoB |
| de la Torre, 2020^50^ | Unclear RoB:  Compared populations not homogeneous | Low RoB | Low RoB | Low RoB | Low RoB | Low RoB | Unclear RoB:  Selective reporting outcomes | Unclear RoB |
| Giraud, 2021^51^ | Low RoB | Low RoB | Low RoB | Low RoB | Low RoB | Low RoB | Unclear RoB:  Selective reporting outcomes | Unclear RoB |
| Izumi, 2021^52^ | Unclear RoB:  Selection bias | Low RoB | Low RoB | Low RoB | Low RoB | Unclear RoB:  Low MTX dose | Low RoB | Unclear RoB |
| Marsman, 2021^53^ | Low RoB | Low RoB | Low RoB | Low RoB | Low RoB | High RoB:  GC dose not defined | Unclear RoB:  Selective reporting outcomes | High RoB |
| **GCA** | | | | | | | | |
| Nesher, 1997^54^ | Low RoB | Low RoB | Low RoB | Low RoB | Low RoB | Unclear RoB:  pre-defined outcomes very broad (“efficacy of GC dosages”) | Unclear RoB:  Selective reporting outcomes | Unclear RoB |
| Hayreh, 2003^55^ | Low RoB | Low RoB | Low RoB | Low RoB | Low RoB | Low RoB | Low RoB | Low RoB |
| Nesher, 2004^56^ | Unclear RoB:  No complete information regarding some variables: smoking, hyper-homocystinemia, antiphospholipid antibodies, and the use of statins. | Low RoB | Low RoB | Low RoB | Low RoB | Low RoB | Low RoB | Unclear RoB |
| Garcia Martinez, 2004^57^ | Unclear RoB:  Different types and dosages of statins | Low RoB | Low RoB | Low RoB | Low RoB | Low RoB | Low RoB | Unclear RoB |
| Alba, 2014^58^ | Low RoB | Low RoB | Low RoB | Low RoB | Low RoB | Low RoB | Low RoB | Low RoB |
| Les, 2015^59^ | Unclear RoB:  Bias related to clinical reasons of physician in choosing medium dosage or high dosage GC regimen. | Unclear RoB:  Higher rate of ocular ischaemic events at baseline in the HD group with the tendency among physicians towards using a slow-tapering GC in these patients with the most severe forms of GCA. | Low RoB | Low RoB | Low RoB | Low RoB | Low RoB | Unclear RoB |
| Czihal, 2015^60^ | Unclear RoB:  Potential heterogeneity of GC tapering: it seems possible that physician perception of the disease extent may have affected the decision to use steroid-sparing agents | Unclear RoB:  Over-representation of patients suffering from extracranial arterial involvement | Low RoB | Low RoB | Low RoB | Low RoB | Low RoB | Unclear RoB |
| Carbonella 2016^61^ | Unclear RoB:  Outcomes not clearly specified, low sample size | High RoB:  High heterogeneity in treatment dosage of DMARDs and route of administration | Low RoB | Low RoB | Low RoB | Low RoB | High RoB:  Selective reporting outcomes | High RoB |
| Hocevar, 2016^62^ | Low RoB | Unclear RoB:  Definition of early and late onset GCA not standardized  Selection bias (not included all the patients diagnosed and treated in other departments of the centre because of missing data) | Low RoB | Low RoB | Low RoB | Low RoB | Low RoB | Unclear RoB |
| Labarca, 2016^63^ | Unclear RoB:  Treatment was not standardized and both initial dose and tapering regimens  were at the discretion of the treating physician. | Unclear RoB:  Only biopsy-proven patients were included in this study. | Low RoB | Low RoB | Low RoB | Unclear RoB:  Three types of relapses included (GCA symptoms + increased inflammatory markers, isolated laboratory elevation without other explainable and GCA symptoms with negative inflammatory markers): this may have increased the total number of observed  relapse events. | Low RoB | Unclear RoB |
| Hocevar, 2019^64^ | Unclear RoB:  Small sample size | Low RoB | Low RoB | Low RoB | Low RoB | Unclear RoB:  Not mentioned the definition of relapses | Low RoB | Unclear RoB |
| Calderon-Goercke, 2019^65^ | Low RoB | Unclear RoB:  TCZ was not used in new- onset GCA, but only in refractory patients to conventional therapy. | Low RoB | Low RoB | Unclear RoB:  A considerable percentage of patients lost to follow-up (71%). | Unclear RoB:  The definition of relapse required an increase of acute phase reactants, but TCZ may  reduce serum CRP levels, ESR, and fibrinogen | Low RoB | Unclear RoB |
| Koster, 2019^66^ | Unclear RoB:  Initiation and titration of MTX was not standardized and was at the discretion of the treating  physician. | Unclear RoB:  Higher rate of relapses prior to initiation in patients receiving MTX | Low RoB | Low RoB | Low RoB | Low RoB | Low RoB | Unclear RoB |
| Tengesdal, 2019^67^ | Unclear RoB:  Low sample size.  Baseline differences among MTX-treated and LEF-treated patients despite being non-significant. | Low RoB | Low RoB | Low RoB | Low RoB | Low RoB | Low RoB | Unclear RoB |
| Ponte, 2020^68^ | Low RoB | Low RoB | Low RoB | Low RoB | Low RoB | Low RoB | Low RoB | Low RoB |
| Calderon-Goercke, 2021^69^ | Low RoB | Low RoB | Low RoB | Low RoB | Unclear RoB:  Only 53% of patients completed the follow-up at first year | Unclear RoB:  The definition of relapse required an increase of acute phase reactants, but TCZ may  reduce serum CRP levels, ESR, and fibrinogen | Low RoB | Unclear RoB |
| Clement, 2021^70^ | Unclear RoB:  Small cohort.  Factors associated with relapses determined post hoc. | Low RoB | Low RoB | Low RoB | Low RoB | Low RoB | Low RoB | Unclear RoB |
| Rossi, 2021^71^ | High RoB:  Heterogeneity in previous treatment with csDMARDs and GC dosages before bDMARD introduction (TCZ vs ABA) | Low RoB | Low RoB | Low RoB | Low RoB | Unclear RoB:  pre-defined outcomes very broad (“efficacy of DMARDs”) | Unclear RoB:  Selective reporting outcomes | High RoB |
| Schönau, 2021^72^ | Low RoB | Unclear RoB:  Baseline prevalence of cranial symptoms related to GCA was indeed higher in the tocilizumab-treated group than in the two other groups. | Low RoB | Low RoB | Low RoB | Low RoB | Low RoB | Unclear RoB |
| Tomelleri, 2022^73^ | Unclear RoB:  Bias related to clinical reasons of physician in choosing treatment according to disease severity. | Unclear RoB:  Potential misclassification of some patients (10-days delay between GC treatment and PET) | Low RoB | Low RoB | Low RoB | Low RoB | Low RoB | Unclear RoB |
| **PMR and GCA** | | | | | | | | |
| Delecoeuillerie, 1988^74^ | Unclear RoB:  Selection bias | Low RoB | Low RoB | Low RoB | Low RoB | Low RoB | Unclear RoB:  Selective reporting outcomes | Unclear RoB |
| Kyle 1989^75^ | High RoB:  Baseline population characteristic not well described | Low RoB | Low RoB | Low RoB | Low RoB | High RoB:  Pre-defined outcomes not specified | High RoB:  Selective reporting outcomes | High RoB |
| Kyle 1989^76^ | High RoB:  Baseline population characteristic not well described | Low RoB | Low RoB | Low RoB | Low RoB | High RoB:  Pre-defined outcomes not specified | High RoB:  Selective reporting outcomes | High RoB |

**Supplementary Table S4.** Outcomes and treatment targets in RCTs on PMR

**Legend**. Green headings: low risk of bias (RoB); yellow headings: unclear RoB; red headings: high RoB.

**Abbreviations**: PMR, polymyalgia rheumatica; D, day; W, week; M, month; N, number; NS, non-significant; TCZ, tocilizumab; PDN, prednisone; GC, glucocorticoids; MTX, methotrexate; RTX, rituximab; PBO, placebo; AEs, adverse events; CRP, C-reactive protein; ESR, erythrocyte sedimentation rate; TNF-a, tumor necrosis factor alpha; IL-6, interleukin 6; ACTH, adrenocorticotropic hormone; MR, modified release; IR, immediate release; PPP, per protocol population; BMC, bone mineral content; BMD, bone mineral density.

| Author, year | Population | N. of patients (women %) | Intervention | Control group | Outcome / Treatment target(s) | Main findings |
| --- | --- | --- | --- | --- | --- | --- |
| Bonelli, 2022^1^ | PMR, new | 36 (53%) | 16 W sc TCZ 162 mg QW + 11 W oral PDN | PBO + 11W PDN | **1) GC-free remission at W16, W12 and W24**  **2) Time to first relapse, cumulative PDN dose at W16 and W24, AEs** | GC-free remission at W 16: 63% in the TCZ vs 11.8% in the placebo group (p=0.002); GC-free remission at w 12 and 24: 57.9 vs 18% (p=0.02 for both). Time to first relapse: 130 vs 82 d (p=0.007). Cumulative PDN dose at w 16: 727 vs 935 (p=0.003); at w 24: 781 vs 1290 (p=0.001) |
| Caporali, 2004^2^ | PMR, new, untreated | 72 (67%) | 48 W oral MTX 10 mg QW + 24W PDN | PBO QW + 24W PDN | **1) GC withdrawal at W76, W24 and W48**  **2) N of relapses; GC duration, cumulative GC dose; AEs** | Patients with ≥1 relapse at W 24-48 (MTX vs PNDN: 31 vs 63%; p = .02; W 76: 47 vs 73%; p = 0.04. Cumulative GC dose W 76: 2.1 g vs 3.0 g; p = 0.003. Discontinuation of GC: W 48: 81 vs 47%; p = .008; W 76: 88 vs 53%; p = 0.003. |
| Cutolo, 2017^3^ | PMR, new, untreated | 62 (69%) | 4W 15 mg modified release PDN at 22:00±30 min | 4W 15 mg immediate-release PDN between 5:00 and 9:00 am | **1) % of complete responders at W4 and**  **2) Δ in PMR VAS score, duration of morning stiffness, % of patients with improvement ≥70%; CRP, ESR, IL-6; HAQ-DI score, SF-36 domain scores and EQ-5D; AEs** | Complete response at W4: 54% in MR prednisone vs 41% in IR prednisone group.  Non-inferiority of MR versus IR prednisone not proven in the primary PPP analysis (treatment difference: 12.22% in favor of MR prednisone; 95% CI −16% to 40%)  Sensitivity analysis on the full analysis population showed an evident trend favoring MR prednisone (N=62; treatment difference:  16%; 95% CI −9% to 40%). |
| Dasgupta, 1998^4^ | PMR, new, untreated | 60 (72%) | 80W Q3/Q4W intramuscular methylprednisolone | 80W Oral PDN | **1) Remission rate at W12, 48 and 96**  **2) GC cumulative dose at W 12, W48 and W96; changes (from baseline to W12, 48 and 96) in morning stiffness, pain VAS, ESR; GCs-related AEs** | Cumulative glucocorticoid dose at W 96: 2.0 g vs 3.5 g; p < 0.001  GC AEs at W 96: weight gain 0.8 kg vs 3.4 kg; p < 0.001  No difference between groups:  Remission at W 12: 60 vs 67%  Remission at W 96 33 vs 30%  Discontinuation of glucocorticoids at W 96: 33 vs 47% |
| Marsman, 2021^5^ | PMR, new/relapsing | 49 (51%) | RTX iv 1000 mg + 17W PDN | PBO + 17W PDN | **1) GC-free remission (PMR activity score) at W21 after infusion of RTX**  **2) < 5 mg/d PDN, cumulative GC dose at W21; changes (baseline to W21) in ESR and CRP, B-cells; changes in symptoms and PROs; proportion of patients who relapsed; AEs** | GC-free remission at W 21: 48% in the rituximab group vs 21% in the placebo group (difference 27% [one-sided 95% CI 4]; relative risk 2·3 [1·1]; p=0.049); mean change in PMR activity score of –13.8 vs –3·8, p=0.018.  PN < 5 mg/d: 100% vs 54% (absolute difference 46% [one-sided 95% CI 20], relative risk 1·8 [1·3]; p=0.0012. |
| Salvarani, 2007^6^ | PMR, new, untreated | 51 (61%) | 22W Infliximab, 3 mg/kg + 16W oral PND, 15 mg/d | PBO + 16W oral PDN, 15 mg/d | **1) Patients without relapse/recurrence at W52 and through W22**  **2) GC-free patients at W22 and W52; duration of PDN therapy, cumulative GC dosage; N of relapses W52; AEs** | No difference between groups at W 52: Patients without a relapse 30% vs 37%.  Discontinuation of GC: 50% vs 54%  Duration of GC therapy: 26 W vs 22 W  Cumulative GC dose: 1.7 g vs 1.2 g |
| Di Munno, 1995^7^ | PMR, new, untreated | 31 (68%) | 12W daily or every other day deflazacort (DFL) | 12W daily or every other day 6MP | **1) Clinical efficacy (reduction of limb gridle pain, morning stiffness, ESR, CRP, fibrinogen, steroid dosage) at W2, 4, 6 and 12**  **2) AEs** | No differences between groups (p= NS). Both regimens led to clinical improvement over baseline at w 2, 4, 6 and 12 (p < 0.05). |
| Kreiner, 2010^8^ | PMR, new, untreated | 22 (NA) | 2W Etanercept, 25 mg twice per W | 2W Placebo, twice per W | **1) Changes in PMR-AS at W2,**  **2) Changes in ESR, plasma TNF-a, IL-6 concentrations; cumulative intake of tramadol** | No difference between groups at W 2: PMR activity score (32 vs  31 points) |
| Krogsgaard, 1996^9^ | PMR, new, untreated | 30 (63%) | Deflazacort 24 mg/d for 6W then tapered | PDN 20 mg/d for 6W, then tapered | **1) Effects of GC on bone mass at M3, 6 and 12**  **2) Resolution of muscular pain, muscular tenderness, morning stiffness at D6, W6 and M3, 6 and 12; ESR and fibrinogen values at D6, W6 and M3, 6 and 12** | No calcium sparing properties of  deflazacort compared with prednisolone at M12  At month 6: Loss in lumbar BMC correlated to the cumulative dose of GC (r = 0.4; p=0.05). |
| Viapiana, 2015^10^ | PMR, new, untreated | 52 (74%) | 6MP, starting dose 20 mg/d | PDN, staring dose 25 mg/d | **1) Remission rate at W2 and time to achieve remission**  **2) Cumulative CG dosage (M12), duration of GC treatment, time interval for each dose tapering; ESR, CRP, fibrinogen, serum cortisol and ACTH** | Remission at W 2: 100 % of the patients on 6MP vs 89 % of the patients on PN. The mean time to achieve full remission after the first dose was significantly (p < .05) longer for PN (20.3 days) than for 6 MP (15.2 days). |
| Ferraccioli, 1996^11^ | PMR, new | 24 (92%) | MTX, 10 mg QW + 24W oral PDN, 25 mg | 24W oral PDN, starting dose 15 mg | **1) Remission and relapse rate at M12**  **2) BMD; GC cumulative dose, GC discontinuation; AEs** | Patients with ≥1 relapse at M 12: 50 vs 100% (p = 0.013)  Discontinuation of GC at M 12: 50 vs 0% (p = 0.013)  Cumulative GC dose at M 12: 1.8 g vs 3.2 g (p= 0.0001) |

**Supplementary Table S5.** Treatment targets and outcomes in RCTs on GCA

**Legend.** Green headings: low risk of bias (RoB); yellow headings: unclear RoB; red headings: high RoB, black headings: indetermined RoB.

**Abbreviations:** giant cell arteritis (GCA), week (W), month (M), days (d), glucocorticoid (GC), prednisone (PDN), adverse events (AEs), tocilizumab (TCZ), placebo (PBO), every week (QW), every other week (EOW), acute ischemic optic neuropathy (AION), patient-reported outcomes (PROs), 36-Item Short-Form Health Survey (SF-36), Physical Component Summary (PCS), Mental Component Summary (MCS) scores, Functional Assessment of Chronic Illness Therapy (FACIT)-Fatigue and Patient Global Assessment of Disease Activity (PtGA), methotrexate (MTX), erythrocyte sedimentation rate (ESR), C-reactive protein (CRP), mavrilimumab (MAV), hazard ratio (HR), odds ratio (OR), arthritis impact measurement scales (AIMS), magnetic resonance angiography, abatacept (ABA), adalimumab (ADA), infliximab (IFX), hydroxychloroquine (HCQ), secukinumab (SEC), sirukumab (SIR), treatment-emergent adverse events (TEAE), bone mineral density (BMD), bone mineral content (BMC), female to male ratio (F/M), not reported (NR), cyclosporine A (CsA).

| Author, year | Population | N. of patients (women %) | Intervention | Control group | Outcome / Treatment target(s) | Main findings |
| --- | --- | --- | --- | --- | --- | --- |
| Stone 2017  (GiACTA double blind phase)^12^ | New-onset and relapsing GCA | n = 250  (74.9 %) | TCZ sc 162 mg QW or Q2W + 26W PDN | PBO + 26W PDN or 52W PDN | **1) Sustained GC-free remission at W52**  **2) Cumulative GC dosage at W52; serious AEs^12^** | Sustained GC-free remission at W52 higher in TCZ QW/EOW vs PBO + PDN tapered in 26 W or W52: 56 % vs 53 % vs 14% vs 18%, all p < 0.001  Cumulative GC dose lower in TCZ groups vs PBO: 1862 mg in each TCZ group vs 3296 mg in PBO + 26W taper vs 3818 mg in PBO + 52W taper, all p<0.001  Rate of serious AEs: 15% of TCZ QW recipients vs 14% of TCZ Q2W vs 22% of PBO + 26W taper vs 25% of PBO + 52W taper. |
|  |  |  |  |  | **1) PROs evaluated by questionnaires (SF-36, PCS, MCS, FACIT, PtGA)^15^** | At W52, PCS and MCS scores improved with TCZ-QW + PDN-26 but worsened in both PBO + PDN groups (p < 0.001)  TCZ-QW + PDN-26 improved 4/8 SF-36 domains compared with PBO + PDN-26 and 6/8 domains compared with PBO + PDN-52 (p < 0.01). |
|  |  |  |  |  | **1) Sustained remission at W52^19^**  **2) Mean annual relapse rate^19^** | 6/14 (43%) of TCZ + MTX patients achieved sustained remission vs 76/135 (56%) treated with TCZ without MTX vs 0% of the 14 PBO + MTX-treated vs 16/87 (18%) PBO without MTX- treated  Non-significant difference in the relapse rate at W52 in MTX-treated vs MTX-untreated in TCZ recipients: 0.76 with MTX vs 0.47 without MTX; p = 0.2549 and among PBO recipients: 1.89 vs 1.46; p = 0.4611. |
|  |  |  |  |  | **1) PDN dosage at the time of flare until W52**  **2) CRP and ESR levels at the time of flare until W52^17^** | 23/36 (64%) TCZ recipients relapsed while still receiving PDN 2 mg/day vs 45/59 (76%) of PBO recipients receiving PDN 5mg/day, p not reported.  33 flares (92%) in TCZ-treated groups and 20 (34%) in PBO + Pred-treated groups occurred with normal CRP levels. More than half of the PBO + Pred-treated patients had elevated CRP without flares.  Among patients starting PDN ≤30 mg/day, the risk for flare was significantly lower among TCZ -treated patients than PBO+PDN -26W patients (HR, 0.21 [99% CI, 0.08 -0.54; p < 0.0001] for TCZ -QW+PDN -26 and 0.28 [0.09 -0.86; p = 0.0035] for TCZ -Q2W+PDN -26). |
|  |  |  |  |  | **1) Sustained remission rate at W52**  **2) Rate of flares and time to flare, Cumulative GC dose and AEs^18^** | Sustained remission at W52 higher in TCZ recipients vs PBO in all 3 groups (PMR only, 45.2% vs 19.0%, P = 0.0446; cranial only, 60.3% vs 19.4%, P = 0.0001; PMR and cranial, 55.0% vs 11.4%, P < 0.0001).  Lower flare rates in TCZ vs PBO for patients with cranial symptoms only and both symptoms (respectively, 0.40 vs 0.93, p = 0.0138 and 0.51 vs 2.27, p < 0.0001).  In all subgroups, lower rate of flares in TCZ-treated vs PBO (PMR only, 41.9% vs 57.1%; cranial only, 20.7% vs 47.2%; PMR and cranial, 31.7% vs 81.8%). |
|  |  |  |  |  | **1) Treatment failure risk**  **(Inability to achieve remission by W12 or relapse between W12 and 52)^16^** | Treatment failure less likely in the TCZ/PDN group than the PBO/PDN group (OR, 0.2; 95%CI, 0.1 to 0.3; p<0.0001). |
| Stone 2021^14^  (GiACTA open extension) | New-onset and relapsing GCA | n = 250  (74.9 %) | TCZ sc 162 mg QW or Q2W + 26W PDN | PBO + 26W PDN or 52W PDN | **1) Maintenance of TCZ-free and GC-free remission after 2 years of TCZ and GC discontinuation at W52^14^**  **2) Cumulative GC dosage at third year; time to achieve remission in relapsing patients when restoring TCZ alone; TCZ + GC or GC alone; AEs^14^** | 25/59 (42%), entering part 2 and assigned to TCZ, maintained TCZ-free and GC-free clinical remission vs 8/28 (29%) of TCZ Q2W recipients vs 7/12 (58%) of PBO + 26W PDN vs 10/16 (63%) of PBO + 52W taper, p not reported.  Cumulative GC dosage: 2647 mg for TCZ QW vs 3948 mg for TCZ Q2W vs 5277 mg for PBO +26W PDN vs 5323 mg for PBO +52W PDN (p < 0.01 for TCZ QW vs PBO and p<0·05 for TCZ Q2W vs PBO groups).  No new or unexpected safety findings were reported over the full 3 years of the study. |
|  |  |  |  |  | **1) Time to first flare over 3 years^77^** | Time to first flare: 576 days in TCZ QW vs 454 days in TCZ EOW vs 202 days in PBO-treated patients, p not reported.  Rate of flares over 3 years: new-onset, 24 (51%) vs relapsing 28 (53%) in TCZ QW, new-onset 19 (73%) vs relapsing 15 (65%) in TCZ EOW and new-onset 33 (72%) vs 38 (69%) in the PBO group, p not reported. |
| Cid 2022^78^ | GCA patients in GC-induced remission | n = 70  (71%) | MAV 150 mg sc Q2W + 26W PDN | PBO sc Q2W + 26W PDN | **1) Time to flare by W26**  **2) Sustained remission at W26; cumulative GC dosage at W26; Time to elevated ESR and CRP by W26; Percentage of patients completing glucocorticoid taper and with no signs or symptoms of GCA at W26; AEs** | MAV reduced the risk of flare vs PBO (HR, 0.38; 95%CI 0.15 to 0.92; p=0.026).  Median time to flare was 25.1 weeks in the PBO group, but the median was not reached in the MAV group (not estimable because of the few events).  In MAV recipients, flares occurred in 19% (n=8) vs PBO recipients displaying flares in 46% (n=13).  Sustained remission at W26 was 83% for MAV vs 50% for PBO recipients (p=0.0038).  Cumulative GC dosage at W26 in MAV vs PBO: 2074 mg vs 2403 mg, p = 0.067  Percentage of patients completing GC taper and with no signs or symptoms of GCA by W26: 30 (71.4%) vs 9 (32.1%), p = 0.0003  AEs: 78.6% (n=33) of MAV and 89.3% (n=25) of PBO. No deaths or vision loss occurred in either group. |
| Mazlumzadeh 2006^21^ | New onset GCA (treated for < 10 days with GC) | n = 27  (70%) | IV pulse methylprednisolone (15 mg/kg/daily) + oral GC 40 mg/day in tapering until control of disease activity | IV saline + oral GC 40 mg daily with same tapering | **1) Rate of patients taking PDN ≤ 5 mg daily at W36**  **2) Sustained remission at W36, W52 and W78; rate of patients discontinuing GC treatment at W78; cumulative PDN dose; number of relapses; GC-related AEs** | A higher rate of patients taking daily PDN ≤ 5 mg daily at W36 IV pulse methylprednisolone-treated vs controls: 10/14 (71 %) vs 2/13 (15%), P = 0.003.  Higher number of sustained remissions after discontinuation of treatment in the IV GC–treated group and a lower median daily dose of prednisone at 78 weeks: 85.7% vs 33.3%, p = 0.0004  The median cumulative dose of oral prednisone, excluding the IV GC dose, was 5,636 mg in the IV GC–treated group compared with 7,860 mg in the IV saline–treated group (P = 0.001). |
| Spiera 2001^22^ | New onset GCA in GC treatment ≤ 1-month | n = 21  (72%) | MTX 7.5 mg/week + oral PDN | PBO + oral PDN | **1) Cumulative GC dosage at W52**  **2) Total duration of GC treatment, time to reach a 10mg daily PDN dose at first year, number of flares** | Non-significant difference in the cumulative GC dosage at first year between MTX-treated combined with oral PDN compared with PBO + oral PDN, 6.5 g vs 5.9 g, p = 0.5  Duration of GC treatment: 68W vs 60W  Cumulative GC dose: 6.5 g vs 5.9 g  Quality of life: similar SF36 and AIMS scores (no data reported)  Comparable number of AEs between the two groups |
| Villiger 2016^23^ | New-onset or recurrent GCA | n = 30  (70%) | TCZ (8 mg/kg) IV Q4W until W52 + oral PDN began at 1 mg/kg and tapered to 0 mg following a predefined scheme | PBO IV Q4W until W52 + oral PDN began at 1 mg/kg and tapered to 0 mg according to a predefined scheme | **1) Rate of complete remission at a PDN dose of 0.1 mg/kg/day at W12.**  **2) Relapse-free survival at W52, cumulative GC dosage, AEs^23^** | Remission at W12 in TCZ-treated patients vs PBO-treated patients: 17 (85%) vs 4 (40%), p = 0.03  Time to relapse: 50Wvs 25W; p < 0.001  Discontinuation of GC (52W): 16 (80%) vs 2 (20%); p = 0.004,  Time to discontinuation of GC: 38W vs 50W; p < 0.001  Cumulative GC dose at 12W: 34 mg/kg vs 36 mg/kg; p = .0048, At W26: 41 mg/kg vs 66 mg/kg; p = 0.002, At W52: 43 mg/kg vs 110 mg/kg; p = 0.0005  TCZ-related AEs (W52): Infections: 10 vs 1 event, cardiovascular disease: 1 vs 5 events, Neutropenia: 9 vs 0 events, Leukopenia: 15 vs 1 events. No Relapse-free survival at W52: 17 (85%) TCZ treated patients and 2 (20%) in the PBO group (p = 0.001) |
|  |  |  |  |  | **1) Rate of patients in remission on MRA (LVV MRA score of 0 = no mural thickening, no enhancement or 1= no thickening, slight mural enhancement) at W12 (GC dose of 0.1 mg/kg/d)^24^**  **2) Number of patients with complete MRA remission at W52 and the change in the vasculitis score^24^.** | Three (33%) out of 9 TCZ patients showed normalization of vessel wall signals on MRA at W12 compared with one (25%) of 4 PBO patient.  At W52, there was additional improvement, but no complete remission, on MRA in 3 TCZ + GC-treated patients, resulting in a median change in the vasculitis score of -1.0, and no improvement in the remaining 2 participants in the placebo + GC group, resulting in a median change in the vasculitis score of -0.5. |
| Langford 2017^25^ | New-onset or relapsing GCA | n = 41  (90%) | ABA IV 10 mg/kg at day 1, 15, 29 and W8, patients in remission at W12 received ABA monthly with daily PDN discontinued at W28 | IV ABA 10 mg/kg at day 1, 15, 29 and W8, patients in remission at W12 received PBO monthly with daily PDN discontinued at W28 | **1) Rate of patients free from relapses at W52**  **2) Duration of remission, AEs** | Relapse-free survival at W52: 48 % of ABA recipients vs 31% of PBO-treated patients, p = 0.049.  Duration of remission: 9.9 months ABA versus 3.9 months PBO; p = 0.023  No difference in the frequency or severity of adverse events, including infection, between the treatment arms. |
| Seror 2014^26^ | New-onset GCA | n = 70  (74.3%) | ADA 40 mg EOW for 10 weeks + PDN regimen (starting dose 0.7 mg/kg/day) | PBO sc EOW for 10 weeks + PDN regimen (starting dose 0.7 mg/kg/day) | **1) Rate of patients in remission on less than 0.1 mg/kg of PDN at W26.**  **2) Rate of relapse-free patients at 12 months, AEs** | Rate of patients in remission at W26: 20 (58.9%) of ADA recipients vs 18 (50%) in PBO (p = 0.46, ns).  The mean PDN daily dose decreased to 0.10 mg/kg (±0.1) in both groups at W52, p = ns  Rate of relapses at W52 in the ADA group vs PBO: 76.0% vs 71.8%, p = ns |
| Hoffman 2002^27^ | New-onset GCA | n = 98  (71%) | GC 1 mg/kg/day (max 60 mg) + oral MTX at a dosage of 0.15 mg/kg/week, increasable up to 15 mg/week MTX | GC 1 mg/kg/day (max 60 mg) + oral PBO | **1) Incidence of treatment failure and disease relapses at W52**  **2) Cumulative GC dosage, duration of GC treatment and AEs** | Incidence of treatment failure at W52: 57.5% in MTX recipients) vs 77.3% in the PBO group, p = 0.26)  Rate of relapses among MTX-treated patients and PBO-patients: 31/51 (61%) vs 31/47 (66%), p = 0.31.  Non-significant differences between groups regarding abnormal elevations of the ESR following initial remissions, serious morbidity due to GCA, cumulative GC dose, or AEs. In the MTX group, there were fewer cases of GCA relapse heralded by symptoms of isolated polymyalgia rheumatica (1 case versus 5 in the PBO group; P = 0.05). |
| Jover 2001^28^ | New onset GCA | n = 42  (71%) | 10 mg MTX weekly, given from the start of GC treatment, for 24 months | 10 mg PBO weekly, given from the start of GC treatment, for 24 months | **1) Number of relapses at 24 months**  **2) Cumulative GC dosage at 24 months, AEs** | Rate of relapses at 24 months in MTX vs PBO: 9/20, 45% vs. 16/19 84.2%; p = 0.02  Relapse free patients at 24 months in MTX vs PBO: 11/20, 55 % vs 3/19, 16%, p = 0.004.  Mean cumulative PDN: 4187 ± 1529 mg in the MTX group and 5489.5 ± 1396 mg in the PBO group, p = 0.009).  Overall, the rate and severity of AEs were similar between group (p > 0.1 for each comparison). |
| Hoffman 2007^29^ | New onset GCA in GC-induced remission | n = 44  (80%) | GC + IFX, 5 mg/kg at W0, 2, and 6 and every 8 weeks thereafter | GC + PBO at W0, 2, and 6 and every 8 weeks thereafter | **1) Rate of remission free from relapses at W22 and AEs**  **2) Time to first flare, biomarkers, cumulative GC dose and rate of patients who remained relapse-free while the GC dosage was tapered to 10 mg/d.** | Rate of IFX patients in remission vs PBO at W22: 43% vs. 50%, p = 0.65)  Rate of patients tapering PDN to 10 mg/day without relapses: 61% IFX group vs. 75% PBO, p = 0.31.  Incidence of infections: 71% IFX group vs 56% PBO, p = ns |
| Sailler 2009^30^ | Non-complicated new onset GCA | n = 64  F/M NR | GC treatment + HCQ 400 mg for 96 weeks | GC treatment + PBO for 96 weeks | **1) Remission rate with PDN ≤ 5 mg daily at W96**  **2) Rate of relapses, GC cumulative dosage and AEs** | Remission at W96: 14 HCQ patients (43.7%) vs 21 PBO patients (65%), p= 0.22  Rate of relapses at W96: 20 HCQ patients (62.5%) vs 14 PBO (43.7%), p=0.13  Median cumulative GC dosage: 7146 mg in the HCQ group vs 6687 mg in the PBO group (p=0.9). |
| Venhoff 2022^31^ (TitAIN) | New onset GCA patients (diagnosed within 6 weeks) naïve to biological treatment | n = 52  (71%) | SEC 300 mg QW (5 doses) and every 4 weeks through W48 (last dose) + 26W PDN | PBO QW (5 doses) and every 4 weeks through W48 (last dose) | **1) Rate of sustained remission until W28**  **2) Rate of sustained remission until W52, time to first flare** | Sustained remission until W28: 70.1% SEC patients vs 20.3% PBO; OR (posterior median with 95% credibility interval), 9.31 (3.54-26.29).  Until W52, the proportion of GCA patients in sustained remission were 59.3% (38.8%-77.6%) in SEC group vs 8.0% (1.0%-26.0%) in PBO group.  The median time to first GCA flare after baseline was not reached for GCA patients treated with SEC and was 197.0 (101.0-280.0) days for PBO. |
| Schmidt 2020^32^ | Active GCA without ischemic events in the last 12 weeks | n = 161  (77%) | SIR 100 mg Q2W or 50 mg Q4W + 26W or 52W PDN | PBO Q2W + 26W or 52W PDN | **1) Rate of sustained relapse-free remission at W52**  **2) Flares, cumulative GC dose at W52, PROs, changes in ESR and CRP and AEs** | Sustained relapse-free remission at W52 was not achieved in a high proportion of patients due to early study termination.  In part A, approximately 94% of patients reported at least one TEAE. 19.3% of patients discontinued treatment due to a serious TEAE (n.s differences among the groups, mainly infections). |
| Nordborg 1997^33^ | GC-treated GCA | n = 27  (78%) | PDN given with an initial mean dosage of 40 mg (range 15-60 mg) and tapered on the basis of clinical and lab course + 800 mg oral Clodronate at 1, 3 ,5, 7, 9 and 11M | PDN given with an initial mean dosage of 40 mg (range 15-60 mg) and tapered based on clinical and laboratory course + PBO tablets at the same months | **1) BMD and BMC of the total body after 12M of GC treatment.** | BMD at 12M in clodronate-treated vs PBO patients: 1.07±0.12 vs 1.04±0.13, p = ns  BMC at 12M in clodronate-treated vs PBO patients: 2281±571 vs 2145±51, p = ns. |
| Liozon 1993^34^ | Untreated GCA | n = 46  F/M NR | Dapsone + with an initial dosage of PDN 0.7 mg/kg daily tapered according to clinical and biochemical status. Dapsone was continued for other 3 months after PDN discontinuation | PDN with equal tapering  When achieving remission, taper dosage every 4 weeks and then 1 mg every two weeks until withdrawal  ng of 50 % | **1) Total duration of GC therapy**  **2) Relapses rate after GC discontinuation; recovery treatment rate** | Treatment duration with GC in dapsone vs PDN-treated only patients: 13 m, 20 d vs 14 m and 6 d, p = ns  GC cumulative dosage: not reported data  Number of relapses during GC therapy in dapsone vs PDN-treated only patients: 6 vs 4, p = ns  Number of relapses after GC discontinuation in dapsone vs PDN-treated only patients: 1 vs 7, p < 0.02  Number of recovered patients in dapsone vs PDN-treated only: 8 vs 2, p < 0.02.  Rate of severe AEs (12.5%) in patients treated with dapsone: hematological toxicity (neutropenia, hemolytic anemia, methemoglobinemia), exfoliative dermatitis. |
| Schaufelberger 2006^35^ | New-onset GCA | n = 60  (63%) | CsA, 2.0-3.5 mg/kg + oral PDN | Oral PDN only | **1) GC cumulative dosage at 12M and daily maintenance dosage of GC in each group**  **2) Number of flares and remission rate at 12th month.** | AEs led to early drug discontinuation: 9 (30%) in CsA-treated patients (3 [10%] with hypertension, 6 [20%] with creatinine increment) vs 0 in the control group  No data reported concerning efficacy outcomes (remission, relapse, cumulative GC dose) |
| Kupersmith 1999^36^ | GCA with and without visual loss | n = 22  (71%) | Daily GC (range 40–1000 mg) at diagnosis + 10 mg oral MTX/week at W4-6 of GC treatment | Daily GC (range 40–1000 mg) at diagnosis + weekly oral PBO at W 4–6 of GC treatment | **1) Rate of delayed visual loss and drug-related ocular complications at 12M.**  **2) Changes in visual acuity at 1, 3, 6 and 12M; cumulative GC dosage** | No patients developed late visual loss as GC dose was reduced.  At W52, visual acuity, contrast sensitivity, colour vision, and threshold perimetry were not significantly different from the determinations at 4th –5th week. At 1 year, there were no significant cataractous or glaucomatous changes.  Cumulative GC dosage in MTX-treated patients vs PBO: 6184 mg vs 5436 mg, p = 0.39. |
| Hunder 1975^37^ | New onset GCA | n = 60  (80%) | Group A: PDN 20 mg orally every 8 hours for 5 days, then 15 mg of PDN every 8 hours | Group B: 20 mg PDN orally every 8 hours for 5 days, then 45 mg PDN daily  Group C: 20 mg PDN orally every 8 hours for 5 days, then 90 mg PDN daily | **1) Resolution of symptoms at 4W**  **2) Changes in inflammatory markers**  **AEs at 4W** | Rate of symptoms resolution at 4W: 6/20, 30% group C vs 18/20, 90% group A vs 16/20, 80%, group B, p < 0.01  ESR at W4: 14 mm/h in A vs 21 mm/h in B vs 45 mm/h in C, p < 0.01  Numbers of patients with hypercortisolism: 14/20, 70% group A, 7/20, 35% group B and 0% in group C, P < 0.01 |

**Supplementary Table S6.** Outcomes and treatment targets in RCTs on PMR and GCA

**Legend**. Yellow headings: unclear risk of bias (RoB), red headings: high RoB.

**Abbreviations:** PMR, polymyalgia rheumatica; GCA, giant cell arteritis; D, day; W, week; AZA, azathioprine; PDN, prednisone; GC, glucocorticoids; PBO, placebo; Δ, variation; AEs, adverse events; NS, non-significant.

| Author, year | Population | N. of patients (women %) | Intervention | Control group | Outcome / Treatment target(s) | Main findings |
| --- | --- | --- | --- | --- | --- | --- |
| Van der Veen, 1996^38^ | PMR / GCA, new, untreated | 40 (75%) | Deflazacort 24 mg/D for 6W then tapered | PDN 20 mg/d for 6W, then tapered | **1) GC free remission at any time, GC cumulative dose, duration of GC treatment**  **2) Relapse rate; GCA complications, AEs** | No differences (p= NS) in time to achieve remission, duration of remission, number of relapses and cumulative PN dose. |
| De Silva, 1986^39^ | PMR / GCA | 31 (77%) | Azathioprine (AZA), 150 mg/D + oral PDN | PBO +oral PDN | **1) Δ in mean PN dose at 4W- intervals till W52**  **2) AEs** | Daily GC dose at W52: 1.9 mg vs 4.2 mg; p < 0.05 |

**Supplementary Figure S1.** PRISMA flowchart of the included studies

**Legend.** *The outcomes of interest were those related to treatment since this SLR informed the international task force for the development of treat to target recommendations. Outcomes/predictors not of interest were mainly biomarkers used for research purposes which cannot be assessed routinely in daily clinical practice.


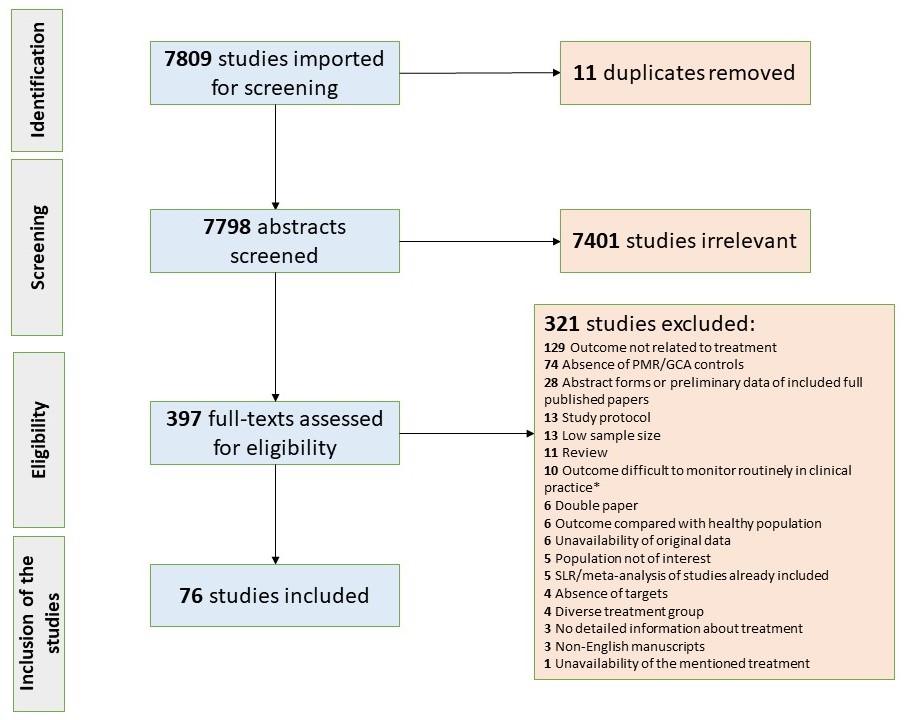


| **Supplementary Box S1.**  Outcomes and treatment targets in non-randomized studies on PMR (PICO 1) |
| --- |

Similar to RCTs, several aspects of treatment were commonly used as outcomes in non-randomized studies in PMR (7/11 studies, 77.8%) including GC discontinuation (3/7, 42.9%), a specific GC target dose (2/7, 28.6%), GC dependency (1/7, 14.3%) or GC cumulative dose (1/7, 14.3%). The second most common outcome addressed was AEs (5/9, 55.6%), while relapse was considered in 4/9 (44.4%) studies only. Remission and laboratory parameters were used in 1 study each (11.1%), while no study investigated PROs.

| **Supplementary Box S2.** Outcomes and treatment targets in non-randomized studies on GCA (PICO 1) |
| --- |

Non-randomised interventional studies

The most frequently reported outcome was the rate of relapses (13/20, 65.0%), followed by treatment related outcomes, in particular the cumulative GC dosage (11/20, 55.0%). The time to reach a specific target PDN dosage and the rate of treatment discontinuation were assessed in 4/20 (20.0%) and 3/20 (15.0%) studies, respectively. Remission and other clinical parameters, such as the rate of clinical improvement/complications (not included in remission and relapse definitions) or the changes of visual acuity, were assessed in 7/20 (35.0 %) and 6/20 (30.0%) of included studies, respectively. Other outcomes concerned laboratory parameters, specifically the variations of inflammatory markers, and general AEs; each reported in 3/20 (15.0%) studies. Imaging outcomes were considered in 2 studies (10.0 %): the ultrasound verified “halo” sign was used in one and the PET-vascular activity score in the other study^68 72^. The necessity to use disease-modifying anti-rheumatic drugs (DMARDs) during follow-up was considered as an outcome in one study (5%)^73^.

| **Supplementary Box S3.** Outcomes and treatment targets in non-randomized studies on mixed populations, PMR + GCA (PICO 1) |
| --- |

Four out of five non-randomized studies (80%) considered treatment-related outcomes in terms of a specific GC target dose (3/4, 75.0%), GC withdrawal (2/4, 50.0%), GC duration (1/4, 25.0%) or GC cumulative dose (1/4, 25.0%). Remission, relapse and AEs were considered in 2/5 (40.0%) studies each, and laboratory parameters (i.e., ESR and CRP) in 1/5 (10.0%). None of the studies evaluated PROs.

| **Supplementary Box S4.** Predictors of outcomes in non-randomized studies on PMR (PICO 8) |
| --- |

Four articles meeting the inclusion criteria and responding to PICO 8 were found. The only study with a low RoB reported an association between the presence of an extracapsular pattern of inflammation in MRI and a better response to treatment (complete GC response and better post-treatment fatigue and function compared to patients without this pattern)^46^. In another study, the musculoskeletal FDG-PET/CT global uptake score at baseline predicted GC dependency at 12 months (with a threshold of 9.5 out of 51 having the best prognostic value)^51^. Among clinical parameters, younger age (<60 years) was associated with greater GC dependency and the need for DMARDs^47^, while low weight at baseline seemed to predict a better short-term response to treatment^44^.

| **Supplementary Box S5.** Predictors of outcomes in non-randomized studies on GCA (PICO 8) |
| --- |

In non-randomized studies, multiple predictors (associated with several outcomes) were retrieved. Three studies (3/7, 42%) assessed the outcomes of GCA patients receiving at diagnosis either a combination of PDN plus conventional synthetic DMARDs, a combination PDN/biological DMARD or GC monotherapy^70 72 73^: Patients treated with a combination therapy yielded a lower rate of relapses^70 73^ and a higher probability to discontinue GC in the long-term^72^.

Two other studies evaluated the use of methylprednisolone pulses and starting with a GC dosage > 40 mg/day as predictors of clinical response. Patients receiving pulse therapy had a shorter time to achieve remission, a higher probability of PDN discontinuation and a shorter time to reach a PDN dose < 5 mg/day compared to patients not receiving this therapy whereas patients receiving, at diagnosis, higher oral GC doses (≥ 40 mg daily) were able to reach a maintenance PDN dosage < 5 mg daily or to discontinue PDN sooner compared with patients starting with lower oral GC dosages (< 40 mg daily)^59 63^

Other candidate predictors investigated were the extent and severity of vascular involvement as verified by ultrasound at baseline: GCA patients with the “halo” sign displayed more frequently jaw claudication and visual symptoms at diagnosis as well as new and persistent visual symptoms at 6^th^ month.^68^ Patients with involvement of several vascular beds (temporal, axillary and subclavian arteries) revealed a higher frequency of relapses and required more often the introduction of an immunosuppressive agent as compared to individuals were only a single vascular site was affected ^60^. In another study, the factors associated with relapses were the introduction of TCZ later than 6 months after diagnosis, absence of ischemic signs at diagnosis, relapse rate > 0.8/year and absence of GC tapering ≤ 5 mg/day before inclusion^70^.

**References**

1. Bonelli M, Radner H, Kerschbaumer A, et al. Tocilizumab in patients with new onset polymyalgia rheumatica (PMR-SPARE): a phase 2/3 randomised controlled trial. *Annals of the Rheumatic Diseases* 2022;24:24. doi: <https://dx.doi.org/10.1136/annrheumdis-2021-221126>

2. Caporali R, Cimmino MA, Ferraccioli G, et al. Prednisone plus methotrexate for polymyalgia rheumatica: a randomized, double-blind, placebo-controlled trial. *Ann Intern Med* 2004;141(7):493-500. doi: 10.7326/0003-4819-141-7-200410050-00005

3. Cutolo M, Hopp M, Liebscher S, et al. Modified-release prednisone for polymyalgia rheumatica: a multicentre, randomised, active-controlled, double-blind, parallel-group study. *RMD Open* 2017;3(1):e000426. doi: 10.1136/rmdopen-2016-000426 [published Online First: 20170317]

4. Dasgupta B, Dolan AL, Panayi GS, et al. An initially double-blind controlled 96 week trial of depot methylprednisolone against oral prednisolone in the treatment of polymyalgia rheumatica. *Br J Rheumatol* 1998;37(2):189-95. doi: 10.1093/rheumatology/37.2.189

5. Marsman DE, den Broeder N, van den Hoogen FHJ, et al. Efficacy of rituximab in patients with polymyalgia rheumatica: a double-blind, randomised, placebo-controlled, proof-of-concept trial. *The Lancet Rheumatology* 2021;3(11):e758-e66. doi: <https://dx.doi.org/10.1016/S2665-9913%2821%2900245-9>

6. Salvarani C, Macchioni P, Manzini C, et al. Infliximab plus prednisone or placebo plus prednisone for the initial treatment of polymyalgia rheumatica: a randomized trial. *Ann Intern Med* 2007;146(9):631-9. doi: 10.7326/0003-4819-146-9-200705010-00005

7. Di Munno O, Imbimbo B, Mazzantini M, et al. Deflazacort versus methylprednisolone in polymyalgia rheumatica: clinical equivalence and relative antiinflammatory potency of different treatment regimens. *J Rheumatol* 1995;22(8):1492-8.

8. Kreiner F, Galbo H. Effect of etanercept in polymyalgia rheumatica: a randomized controlled trial. *Arthritis Res Ther* 2010;12(5):R176. doi: 10.1186/ar3140 [published Online First: 20100920]

9. Krogsgaard MR, Thamsborg G, Lund B. Changes in bone mass during low dose corticosteroid treatment in patients with polymyalgia rheumatica: a double blind, prospective comparison between prednisolone and deflazacort. *Ann Rheum Dis* 1996;55(2):143-6. doi: 10.1136/ard.55.2.143

10. Viapiana O, Gatti D, Troplini S, et al. Prednisone compared to methylprednisolone in the polymyalgia rheumatica treatment. *Rheumatol Int* 2015;35(4):735-9. doi: 10.1007/s00296-014-3105-0 [published Online First: 20140823]

11. Ferraccioli G, Salaffi F, De Vita S, et al. Methotrexate in polymyalgia rheumatica: preliminary results of an open, randomized study. *J Rheumatol* 1996;23(4):624-8.

12. Stone JH, Tuckwell K, Dimonaco S, et al. Trial of Tocilizumab in Giant-Cell Arteritis. *New England Journal of Medicine* 2017;377(4):317-28. doi: 10.1056/NEJMoa1613849

13. Stone JH, Spotswood H, Unizony SH, et al. New-onset versus relapsing giant cell arteritis treated with tocilizumab: 3-year results from a randomized controlled trial and extension. *Rheumatology* 2021;61(7):2915-22. doi: 10.1093/rheumatology/keab780

14. Stone JH, Han J, Aringer M, et al. Long-term effect of tocilizumab in patients with giant cell arteritis: open-label extension phase of the Giant Cell Arteritis Actemra (GiACTA) trial. *The Lancet Rheumatology* 2021;3(5):e328-e36. doi: <http://dx.doi.org/10.1016/S2665-9913%2821%2900038-2>

15. Strand V, Dimonaco S, Tuckwell K, et al. Health-related quality of life in patients with giant cell arteritis treated with tocilizumab in a phase 3 randomised controlled trial. *Arthritis Res Ther* 2019;21(1):64. doi: 10.1186/s13075-019-1837-7 [published Online First: 20190220]

16. Unizony SH, Bao M, Han J, et al. Treatment failure in giant cell arteritis. *Annals of the Rheumatic Diseases* 2021;80(11):1467-74. doi: <https://dx.doi.org/10.1136/annrheumdis-2021-220347>

17. Stone JH, Tuckwell K, Dimonaco S, et al. Glucocorticoid Dosages and Acute-Phase Reactant Levels at Giant Cell Arteritis Flare in a Randomized Trial of Tocilizumab. *Arthritis rheumatol* 2019;71(8):1329-38. doi: 10.1002/art.40876 [published Online First: 20190703]

18. Spiera R, Unizony SH, Bao M, et al. Tocilizumab vs placebo for the treatment of giant cell arteritis with polymyalgia rheumatica symptoms, cranial symptoms or both in a randomized trial. *Seminars in Arthritis & Rheumatism* 2021;51(2):469-76. doi: <https://dx.doi.org/10.1016/j.semarthrit.2021.03.006>

19. Mohan S, Han J, Stone JH. FRI0220 EFFICACY OF ADJUNCTIVE METHOTREXATE IN PATIENTS WITH GIANT CELL ARTERITIS TREATED WITH TOCILIZUMAB PLUS PREDNISONE TAPERING: SUBANALYSIS OF THE GIACTA TRIAL. *Annals of the Rheumatic Diseases* 2020;79(Suppl 1):693-93. doi: 10.1136/annrheumdis-2020-eular.2204

20. Cid MC, Unizony SH, Blockmans D, et al. Efficacy and safety of mavrilimumab in giant cell arteritis: a phase 2, randomised, double-blind, placebo-controlled trial. *Ann Rheum Dis* 2022;81(5):653-61. doi: 10.1136/annrheumdis-2021-221865 [published Online First: 20220309]

21. Mazlumzadeh M, Hunder GG, Easley KA, et al. Treatment of giant cell arteritis using induction therapy with high-dose glucocorticoids: a double-blind, placebo-controlled, randomized prospective clinical trial. *Arthritis Rheum* 2006;54(10):3310-8. doi: 10.1002/art.22163

22. Spiera RF, Mitnick HJ, Kupersmith M, et al. A prospective, double-blind, randomized, placebo controlled trial of methotrexate in the treatment of giant cell arteritis (GCA). *Clin Exp Rheumatol* 2001;19(5):495-501.

23. Villiger PM, Adler S, Kuchen S, et al. Tocilizumab for induction and maintenance of remission in giant cell arteritis: a phase 2, randomised, double-blind, placebo-controlled trial. *Lancet* 2016;387(10031):1921-7. doi: 10.1016/s0140-6736(16)00560-2 [published Online First: 20160304]

24. Reichenbach S, Adler S, Bonel H, et al. Magnetic resonance angiography in giant cell arteritis: results of a randomized controlled trial of tocilizumab in giant cell arteritis. *Rheumatology (Oxford)* 2018;57(6):982-86. doi: 10.1093/rheumatology/key015

25. Langford CA, Cuthbertson D, Ytterberg SR, et al. A Randomized, Double-Blind Trial of Abatacept (CTLA-4Ig) for the Treatment of Giant Cell Arteritis. *Arthritis rheumatol* 2017;69(4):837-45. doi: 10.1002/art.40044 [published Online First: 20170303]

26. Seror R, Baron G, Hachulla E, et al. Adalimumab for steroid sparing in patients with giant-cell arteritis: results of a multicentre randomised controlled trial. *Ann Rheum Dis* 2014;73(12):2074-81. doi: 10.1136/annrheumdis-2013-203586 [published Online First: 20130729]

27. Hoffman GS, Cid MC, Hellmann DB, et al. A multicenter, randomized, double-blind, placebo-controlled trial of adjuvant methotrexate treatment for giant cell arteritis. *Arthritis Rheum* 2002;46(5):1309-18. doi: 10.1002/art.10262

28. Jover JA, Hernández-García C, Morado IC, et al. Combined treatment of giant-cell arteritis with methotrexate and prednisone. a randomized, double-blind, placebo-controlled trial. *Ann Intern Med* 2001;134(2):106-14. doi: 10.7326/0003-4819-134-2-200101160-00010

29. Hoffman GS, Cid MC, Rendt-Zagar KE, et al. Infliximab for maintenance of glucocorticosteroid-induced remission of giant cell arteritis: a randomized trial. *Ann Intern Med* 2007;146(9):621-30. doi: 10.7326/0003-4819-146-9-200705010-00004

30. Mahr A, Hachulla E, de Boysson H, et al. Presentation and Real-World Management of Giant Cell Arteritis (Artemis Study). *Frontiers in Medicine* 2021;8:732934. doi: <https://dx.doi.org/10.3389/fmed.2021.732934>

31. Venhoff N, Schmidt WA, Bergner R, et al. OP0182 SECUKINUMAB IN GIANT CELL ARTERITIS: THE RANDOMISED, PARALLEL-GROUP, DOUBLE-BLIND, PLACEBO-CONTROLLED, MULTICENTRE PHASE 2 TitAIN TRIAL. *Annals of the Rheumatic Diseases* 2022;81(Suppl 1):121-22. doi: 10.1136/annrheumdis-2022-eular.806

32. Schmidt WA, Dasgupta B, Luqmani R, et al. A Multicentre, Randomised, Double-Blind, Placebo-Controlled, Parallel-Group Study to Evaluate the Efficacy and Safety of Sirukumab in the Treatment of Giant Cell Arteritis. *Rheumatol Ther* 2020;7(4):793-810. doi: 10.1007/s40744-020-00227-2 [published Online First: 20200825]

33. Nordborg E, Schaufelberger C, Andersson R, et al. The ineffectiveness of cyclical oral clodronate on bone mineral density in glucocorticoid-treated patients with giant-cell arteritis. *J Intern Med* 1997;242(5):367-71. doi: 10.1046/j.1365-2796.1997.00210.x

34. Liozon F, Vidal E, Barrier J. Does dapsone have a role in the treatment of temporal arteritis with regard to efficacy and toxicity? *Clin Exp Rheumatol* 1993;11(6):694-5.

35. Schaufelberger C, Möllby H, Uddhammar A, et al. No additional steroid-sparing effect of cyclosporine A in giant cell arteritis. *Scand J Rheumatol* 2006;35(4):327-9. doi: 10.1080/03009740500474537

36. Kupersmith MJ, Langer R, Mitnick H, et al. Visual performance in giant cell arteritis (temporal arteritis) after 1 year of therapy. *Br J Ophthalmol* 1999;83(7):796-801. doi: 10.1136/bjo.83.7.796

37. Hunder GG, Sheps SG, Allen GL, et al. Daily and alternate-day corticosteroid regimens in treatment of giant cell arteritis: comparison in a prospective study. *Ann Intern Med* 1975;82(5):613-8. doi: 10.7326/0003-4819-82-5-613

38. van der Veen MJ, Dinant HJ, van Booma-Frankfort C, et al. Can methotrexate be used as a steroid sparing agent in the treatment of polymyalgia rheumatica and giant cell arteritis? *Ann Rheum Dis* 1996;55(4):218-23. doi: 10.1136/ard.55.4.218

39. De Silva M, Hazleman BL. Azathioprine in giant cell arteritis/polymyalgia rheumatica: a double-blind study. *Ann Rheum Dis* 1986;45(2):136-8. doi: 10.1136/ard.45.2.136

40. Gabriel SE, Sunku J, Salvarani C, et al. Adverse outcomes of antiinflammatory therapy among patients with polymyalgia rheumatica. *Arthritis Rheum* 1997;40(10):1873-8. doi: 10.1002/art.1780401022

41. Myklebust G, Gran JT. Prednisolone maintenance dose in relation to starting dose in the treatment of polymyalgia rheumatica and temporal arteritis. A prospective two-year study in 273 patients. *Scand J Rheumatol* 2001;30(5):260-7. doi: 10.1080/030097401753180327

42. Maradit Kremers H, Reinalda MS, Crowson CS, et al. Glucocorticoids and cardiovascular and cerebrovascular events in polymyalgia rheumatica. *Arthritis Rheum* 2007;57(2):279-86. doi: 10.1002/art.22548

43. Cimmino MA, Salvarani C, Macchioni P, et al. Long-term follow-up of polymyalgia rheumatica patients treated with methotrexate and steroids. *Clin Exp Rheumatol* 2008;26(3):395-400.

44. Cimmino MA, Parodi M, Montecucco C, et al. The correct prednisone starting dose in polymyalgia rheumatica is related to body weight but not to disease severity. *BMC Musculoskelet Disord* 2011;12(1):94. doi: 10.1186/1471-2474-12-94 [published Online First: 20110514]

45. Benucci M, Olivito B, Manfredi M, et al. Polymyalgia rheumatica: inflammation suppression with low dose of methylprednisolone or modified-release prednisone. *Eur Rev Med Pharmacol Sci* 2015;19(5):745-51.

46. Mackie SL, Pease CT, Fukuba E, et al. Whole-body MRI of patients with polymyalgia rheumatica identifies a distinct subset with complete patient-reported response to glucocorticoids. *Ann Rheum Dis* 2015;74(12):2188-92. doi: 10.1136/annrheumdis-2015-207395 [published Online First: 20150916]

47. Charpentier A, Verhoeven F, Sondag M, et al. Therapeutic response to prednisone in relation to age in polymyalgia rheumatica: a comparison study. *Clin Rheumatol* 2018;37(3):819-23. doi: 10.1007/s10067-018-3988-3 [published Online First: 20180129]

48. Quartuccio L, Gregoraci G, Isola M, et al. Retrospective analysis of the usefulness of a protocol with high-dose methotrexate in polymyalgia rheumatica: Results of a single-center cohort of 100 patients. *Geriatr Gerontol Int* 2018;18(9):1410-14. doi: 10.1111/ggi.13460 [published Online First: 20180706]

49. Giollo A, Rossini M, Bettili F, et al. Permanent Discontinuation of Glucocorticoids in Polymyalgia Rheumatica Is Uncommon but May Be Enhanced by Amino Bisphosphonates. *J Rheumatol* 2019;46(3):318-22. doi: 10.3899/jrheum.180324 [published Online First: 20181101]

50. de la Torre ML, Rodríguez AM, Pisoni CN. Usefulness of Methotrexate in the Reduction of Relapses and Recurrences in Polymyalgia Rheumatica: An Observational Study. *J Clin Rheumatol* 2020;26(7S Suppl 2):S213-s17. doi: 10.1097/rhu.0000000000001414

51. Giraud N, Prati C, Wendling D, et al. Prognostic value of 18F-fluorodeoxyglucose PET-CT score at baseline on the therapeutic response to prednisone in patients with polymyalgia rheumatica. *Joint Bone Spine* 2021;88(1):105093. doi: <https://dx.doi.org/10.1016/j.jbspin.2020.105093>

52. Izumi K, Murata O, Higashida-Konishi M, et al. Steroid-Sparing Effect of Tocilizumab and Methotrexate in Patients with Polymyalgia Rheumatica: A Retrospective Cohort Study. *Journal of Clinical Medicine* 2021;10(13):30. doi: <https://dx.doi.org/10.3390/jcm10132948>

53. Marsman D, Bolhuis T, Broeder ND, et al. Effect of add-on methotrexate in polymyalgia rheumatica patients flaring on glucocorticoids tapering: a retrospective study. *Rheumatology International* 2021;41(3):611-16. doi: <https://dx.doi.org/10.1007/s00296-020-04783-2>

54. Nesher G, Rubinow A, Sonnenblick M. Efficacy and adverse effects of different corticosteroid dose regimens in temporal arteritis: a retrospective study. *Clin Exp Rheumatol* 1997;15(3):303-6.

55. Hayreh SS, Zimmerman B. Visual deterioration in giant cell arteritis patients while on high doses of corticosteroid therapy. *Ophthalmology* 2003;110(6):1204-15. doi: 10.1016/s0161-6420(03)00228-8

56. Nesher G, Berkun Y, Mates M, et al. Low-dose aspirin and prevention of cranial ischemic complications in giant cell arteritis. *Arthritis Rheum* 2004;50(4):1332-7. doi: 10.1002/art.20171

57. García-Martínez A, Hernández-Rodríguez J, Grau JM, et al. Treatment with statins does not exhibit a clinically relevant corticosteroid-sparing effect in patients with giant cell arteritis. *Arthritis Rheum* 2004;51(4):674-8. doi: 10.1002/art.20541

58. Alba MA, García-Martínez A, Prieto-González S, et al. Treatment with angiotensin II receptor blockers is associated with prolonged relapse-free survival, lower relapse rate, and corticosteroid-sparing effect in patients with giant cell arteritis. *Seminars in Arthritis and Rheumatism* 2014;43(6):772-77. doi: <https://doi.org/10.1016/j.semarthrit.2013.10.009>

59. Les I, Pijoán JI, Rodríguez-Álvarez R, et al. Effectiveness and safety of medium-dose prednisone in giant cell arteritis: a retrospective cohort study of 103 patients. *Clin Exp Rheumatol* 2015;33(2 Suppl 89):S-90-7. [published Online First: 20150526]

60. Czihal M, Piller A, Schroettle A, et al. Impact of cranial and axillary/subclavian artery involvement by color duplex sonography on response to treatment in giant cell arteritis. *J Vasc Surg* 2015;61(5):1285-91. doi: 10.1016/j.jvs.2014.12.045 [published Online First: 20150203]

61. Carbonella A, Berardi G, Petricca L, et al. Immunosuppressive Therapy (Methotrexate or Cyclophosphamide) in Combination with Corticosteroids in the Treatment of Giant Cell Arteritis: Comparison with Corticosteroids Alone. *J Am Geriatr Soc* 2016;64(3):672-374. doi: 10.1111/jgs.14004

62. Hocevar A, Rotar Z, Jese R, et al. Do Early Diagnosis and Glucocorticoid Treatment Decrease the Risk of Permanent Visual Loss and Early Relapses in Giant Cell Arteritis: A Prospective Longitudinal Study. *Medicine (Baltimore)* 2016;95(14):e3210. doi: 10.1097/md.0000000000003210

63. Labarca C, Koster MJ, Crowson CS, et al. Predictors of relapse and treatment outcomes in biopsy-proven giant cell arteritis: a retrospective cohort study. *Rheumatology (Oxford)* 2016;55(2):347-56. doi: 10.1093/rheumatology/kev348 [published Online First: 20150918]

64. Hočevar A, Ješe R, Rotar Ž, et al. Does leflunomide have a role in giant cell arteritis? An open-label study. *Clin Rheumatol* 2019;38(2):291-96. doi: 10.1007/s10067-018-4232-x [published Online First: 20180806]

65. Calderón-Goercke M, Loricera J, Aldasoro V, et al. Tocilizumab in giant cell arteritis. Observational, open-label multicenter study of 134 patients in clinical practice. *Semin Arthritis Rheum* 2019;49(1):126-35. doi: 10.1016/j.semarthrit.2019.01.003 [published Online First: 20190105]

66. Koster MJ, Yeruva K, Crowson CS, et al. Efficacy of Methotrexate in Real-world Management of Giant Cell Arteritis: A Case-control Study. *J Rheumatol* 2019;46(5):501-08. doi: 10.3899/jrheum.180429 [published Online First: 20190115]

67. Tengesdal S, Diamantopoulos AP, Myklebust G. Leflunomide versus methotrexate in treatment of giant cell arteritis: comparison of efficacy, safety, and drug survival. *Scand J Rheumatol* 2019;48(4):333-35. doi: 10.1080/03009742.2019.1575980 [published Online First: 20190305]

68. Ponte C, Serafim AS, Monti S, et al. Early variation of ultrasound halo sign with treatment and relation with clinical features in patients with giant cell arteritis. *Rheumatology (Oxford)* 2020;59(12):3717-26. doi: 10.1093/rheumatology/keaa196

69. Calderón-Goercke M, Castañeda S, Aldasoro V, et al. Tocilizumab in refractory giant cell arteritis. Monotherapy versus combined therapy with conventional immunosuppressive drugs. Observational multicenter study of 134 patients. *Semin Arthritis Rheum* 2021;51(2):387-94. doi: 10.1016/j.semarthrit.2021.01.006 [published Online First: 20210127]

70. Clément J, Duffau P, Constans J, et al. Real-world Risk of Relapse of Giant Cell Arteritis Treated With Tocilizumab: A Retrospective Analysis of 43 Patients. *J Rheumatol* 2021;48(9):1435-41. doi: 10.3899/jrheum.200952 [published Online First: 20210215]

71. Rossi D, Cecchi I, Sciascia S, et al. An agent-to-agent real life comparison study of tocilizumab versus abatacept in giant cell arteritis. *Clin Exp Rheumatol* 2021;39 Suppl 129(2):125-28. doi: 10.55563/clinexprheumatol/l0hd9v [published Online First: 20210223]

72. Schonau V, Roth J, Tascilar K, et al. Resolution of vascular inflammation in patients with new-onset giant cell arteritis: data from the RIGA study. *Rheumatology* 2021;60(8):3851-61. doi: <https://dx.doi.org/10.1093/rheumatology/keab332>

73. Tomelleri A, Campochiaro C, Sartorelli S, et al. Presenting features and outcomes of cranial-limited and large-vessel giant cell arteritis: a retrospective cohort study. *Scandinavian Journal of Rheumatology* 2022;51(1):59-66. doi: <https://dx.doi.org/10.1080/03009742.2021.1889025>

74. Delecoeuillerie G, Joly P, Cohen de Lara A, et al. Polymyalgia rheumatica and temporal arteritis: a retrospective analysis of prognostic features and different corticosteroid regimens (11 year survey of 210 patients). *Ann Rheum Dis* 1988;47(9):733-9. doi: 10.1136/ard.47.9.733

75. Kyle V, Hazleman BL. Treatment of polymyalgia rheumatica and giant cell arteritis. I. Steroid regimens in the first two months. *Ann Rheum Dis* 1989;48(8):658-61. doi: 10.1136/ard.48.8.658

76. Kyle V, Cawston TE, Hazleman BL. Erythrocyte sedimentation rate and C reactive protein in the assessment of polymyalgia rheumatica/giant cell arteritis on presentation and during follow up. *Ann Rheum Dis* 1989;48(8):667-71. doi: 10.1136/ard.48.8.667

77. Stone JH, Spotswood H, Unizony SH, et al. New-onset versus relapsing giant cell arteritis treated with tocilizumab: 3-year results from a randomized controlled trial and extension. *Rheumatology* 2021;29:29. doi: <https://dx.doi.org/10.1093/rheumatology/keab780>

78. Cid MC, Unizony SH, Blockmans D, et al. Efficacy and safety of mavrilimumab in giant cell arteritis: a phase 2, randomised, double-blind, placebo-controlled trial. *Annals of the Rheumatic Diseases* 2022;81(5):653-61. doi: <https://dx.doi.org/10.1136/annrheumdis-2021-221865>
